# Sustainable process for increased lutein production in green microalga *Coelastrella thermophila*

**DOI:** 10.3389/fmicb.2026.1833061

**Published:** 2026-08-10

**Authors:** Sudipto Adhikary, Sejal Dangolia, Mirnalini Juyal, Rushi Joshi, Debarati Paul, Debashish Ghosh, Shreyansh Jain, Sourish Bhattacharya

**Affiliations:** 1 Applied Phycology and Biotechnology Division, CSIR-Central Salt and Marine Chemicals Research Institute, Bhavnagar, India; 2 Academy of Scientific and Innovative Research (AcSIR), CSIR-HRDC Campus, Ghaziabad, Uttar Pradesh, India; 3 Material Resource Efficiency Division, CSIR-Indian Institute of Petroleum, Dehradun, Uttarakhand, India; 4 Intrinsic Foundries Inc., Boston, MA, United States; 5 Intrinsic Foundries, IIOT Innovation Private Limited, Hazaribagh, Jharkhand, India

**Keywords:** *Coelastrella thermophila*, lutein, marine microalgae, nanoparticles, reactive oxygen species

## Abstract

**Introduction:**

*Coelastrella thermophila* represents a highly promising microalgal platform for sustainable lutein production due to its thermotolerance, high biomass productivity, robust carotenoid biosynthetic capacity, and compatibility with carbon capture technologies. The integration of optimized cultivation strategies, advanced bioprocess engineering, and downstream processing technologies is expected to accelerate the commercialization of lutein production from this microalga, contributing to the growing global demand for natural, environmentally friendly carotenoid products.

**Methods:**

*Coelastrella thermophila* P2, isolated from marine brine samples collected at the CSIR-CSMCRI Experimental Salt Farm, was maintained axenically on Bold Basal Medium (BBM) and cultivated using a two-stage strategy to enhance lutein production. Initially, the strain was grown in modified CSMCRI P2 (Zarrouk’s) medium under controlled conditions (16 h light/8 h dark) to maximize biomass accumulation. During the second stage, late logarithmic-phase biomass was transferred to alkaline medium under optimized reactive oxygen species (ROS)-inducing conditions, combining high salinity and elevated pH to stimulate carotenoid biosynthesis. Purification of lutein was achieved using Fe_3_O_4_ magnetic nanoparticles as selective adsorbents, followed by magnetic separation and desorption with tetrahydrofuran (THF). The purified lutein was characterized by UV–Vis spectroscopy, nuclear magnetic resonance (NMR), and high-performance liquid chromatography (HPLC), while the synthesized Fe_3_O_4_ nanoparticles were characterized using Fourier-transform infrared spectroscopy (FTIR) and X-ray diffraction (XRD).

**Results and discussion:**

Under optimized conditions, *Coelastrella thermophila* provided a biomass concentration of 1.1 g/L with a highly enriched lutein fraction representing approximately 10% of the total carotenoid fraction, as confirmed by UV-visible spectroscopy, HPLC and NMR. Surprisingly, in the current process, *Coelastrella thermophila* produced only lutein, a carotenoid, at a higher concentration, thereby reducing downstream production costs. Further at downstream, reusable Fe3O4 nanoparticles were used to purify separate lutein from other cellular impurities in a cost-effective manner. This is the first report on biomanufacturing a major single pigment at higher concentration from microalgae, which can provide new insights into microalgal pigment production.

## 1 Introduction

Unicellular microalgae are of great interest for the upcoming biotech era due to their high growth rate and their applications in the biomanufacturing of high-value, commercially important products. Lutein and other carotenoids, including *α* & *β*-carotene, zeaxanthin, violaxanthin, alloxanthin & neoxanthin, are predominantly considered as high-value microalgal products and, as a consequence, studies for enhancing microalgal pigment are always a point of interest. Current market demand for microalgal pigments includes the health care, cosmetics, pharmaceuticals, food, and textile industries. Therapeutic benefits of microalgal pigment molecules include antioxidant and anti-inflammatory responses, provitamin A properties, and protective effects on the retina (Choo et al., 2020; Gauthier et al., 2020). Microalgal pigments are classified into three main classes: Chlorophyll, Carotenoids, and Phycobilin proteins. However, the most common microalgal carotenoid group is xanthophyll, and lutein is a common member of this family. The strongest commercial pigment is astaxanthin, widely marketed as a natural antioxidant. The global astaxanthin market was valued at approximately USD 1.96 billion in 2025 and is projected to exceed USD 4.2 billion by 2033, reflecting strong demand from nutraceutical and preventive healthcare sectors. Nutraceutical applications alone are projected to reach nearly USD 496 million in 2025 and are growing at a Compound Annual Growth Rate (CAGR) of more than 10%^1^. However, the global lutein market in 2025 is approximately USD 350–420 million, growing at 5–6% annually^2^.

Several microalgal species have been adopted as high pigment producers, like *Dunaliella salina* for *β*-carotene and zeaxanthin, astaxanthin from *Chlorella sorokiniana* and *Haematococcus* sp., fucoxanthin from diatoms and *Muriellopsis* sp., phycoerythrin from species of *Porphyridium* and *Anabaena* sp. (Cordero et al., 2011; Eilers et al., 2016; Fu et al., 2014; Tripathi et al., 1999; Pangestuti et al., 2026; Chettri et al., 2026; Xu et al., 2026). Among these, some novel strains of *Chlorella* sp. can produce lutein, zeaxanthin, and violaxanthin in their cells. However, the accumulation of a mixture of carotenoid compounds within microalgal cells makes downstream processing and recovery of the pure compound cost-intensive and limits the productivity of the target pigment. Further, pigment production from microalgae at a commercial scale often becomes challenging due to low yields from a particular strain. However, it is possible to increase the pigmentation using different stress and oxidative stress, which are highly effective for selective microalgal pigmentation (Fu et al., 2014; Minyuk et al., 2017; Shi et al., 2020; Wei et al., 2008; Zuluaga et al., 2017).

Carotenoids are microalgal secondary metabolites that help harness light energy under specific conditions when photosynthetic pigments are insufficient and act more actively under oxidative stress as a protective mechanism against ROS. Structurally, carotenoids are sub-classified into two major classes: carotene and xanthophylls, and they differ in the absence of an oxygen molecule in their structure. Under regular physiological conditions, *β*-carotene is the common carotenoid species; on the other hand, lutein is an oxidized derivative of the common carotene backbone, and the formation of lutein requires a more oxygen-rich environment, which can be provided by external oxidative stress.

Physiological photosynthetic pigments, such as chlorophylls, harvest photon energy in microalgae, but under ROS, they recruit carotenoids to reduce photoinhibition and serve as a backup for damaged photosynthetic units (Cirulis et al., 2013; Xi et al., 2021). These molecules can harvest photons in the presence of excess reactive oxygen, employing self-reduction and subsequent production of oxygenated pigments such as lutein. Cellular responses to oxidative stress can be of two types: enzymatic & non-enzymatic. The non-enzymatic response of microalgae can produce glutathione, ascorbic acids and their derivatives, tocopherols, flavonoids, carotenoids, cyclosporine amino acids (MAA) and proline (Cirulis et al., 2013; Zuluaga et al., 2017).

The two main approaches to establish microalgal pigment production process are (a) initially to increase the maximum carbon flow towards pigment synthesis, avoiding other metabolites, and (b) triggering specific accumulation of target pigment to reduce downstream cost. Most microalgae produce pigments under stress conditions, and common stressors that trigger microalgal pigmentation include high salinity, nutrient starvation, radiation, or high light intensity. Salinity-induced pigment production requires a large amount of expensive, non-recoverable media salts, and, on the other hand, high light intensity itself is energy-intensive. Although, some microalgal species showed an increase in astaxanthin, lutein, and *β*-carotene concentrations under nutrient starvation of sulphur and phosphorus, or sometimes nitrogen, the production of multiple pigments makes these processes costly (Li et al., 2019; Orosa et al., 2001; Sinetova et al., 2006). However, the most significant pigment accumulation factor in microalgal species is the effect of ROS or reactive oxygen species. Generally, molecular oxygen serves as the final electron acceptor in microalgae, but excess concentrations of molecular oxygen produce toxic metabolites and induce oxidative stress (Cirulis et al., 2013; Hamed et al., 2017). This cellular oxidative stress results from an imbalance between prooxidant and antioxidant species within a cell in response to unusual pollutants, imbalanced pH, excessive light intensity, or irradiation (Cheloni and Slaveykova, 2018; Fal et al., 2022). Photosynthetic organisms are likely to employ carotenoids for self-defence against excess ROS due to chlorophyll-mediated cellular damage. Again, in such a scenario, oxygen molecules compete with carbon dioxide and prompt RuBisCO to fix CO_2_, thereby supporting normal growth. Microalgae are photosynthetic organisms that have specific defence mechanisms against ROS, employing enzymes or various pigments. Oxidative stress in microalgal species can be induced by factors such as high or low pH, irradiation, and high metal concentrations or low nutrient availability. Under such oxidative stress, microalgae produce specific secondary metabolites with high antioxidant activity through several complex microcellular reactions, including the Haber-Weiss reaction, the Fenton process, and the electron transport chain (Kehrer, 2000). Although, oxidative stress produces several high-value metabolites, including carotenoids, their production is often challenging because oxidative stress itself reduces growth and biomass productivity. ROS are detrimental to cells most of the time, but ROS stress, when controlled, can induce modifications to lipids, proteins, enzymes, sterols, and other antioxidant molecules in microalgae (Cirulis et al., 2013; León-Vaz et al., 2019; Xi et al., 2021). However, the response to ROS depends on the strain species and class. It may involve an increase in enzymatic antioxidants such as catalase, glutathione peroxidase, and superoxide dismutase, or in non-enzymatic antioxidants such as xanthophylls, flavonoids, and carotenoids. Some researchers have reported a significant increase in the concentrations of *β*-carotene, zeaxanthin, astaxanthin, and lutein under oxidative stress in *Coccomyxa* sp. and *Chlamydomonas acidophila* sp. (Bermejo et al., 2018; Garbayo et al., 2008). However, the exact mechanism of pH-induced oxidative stress and its subsequent pigmentation at the molecular level remains poorly explored.

Lutein has been extensively utilized for colouring foods, medications, cosmetics, and therapeutics. These microalgal pigments are widely used for several health benefits, including food supplements, joint and tendon health, eye care, neurological disorders, and even cardiovascular health (Fernández-Sevilla et al., 2010). Since, the human body cannot produce pigment molecules, reliance on plant- or algal-based sources are required. Lutein & zeaxanthin are the only carotenoid that are absorbed into the bloodstream after consumption and accumulates in the human retina. Microalgal-derived lutein has been reported to have significant health benefits, including maintaining eye health and improving serum levels, and to be negatively correlated with the risk of ocular diseases such as age-related macular degeneration. It also has cell-protective effects by blocking blue light and potentially quenching reactive radicals and inactivating singlet oxygen, thereby acting as an antioxidant (Fernández-Sevilla et al., 2010). Recent studies on microalgal pigments have shown ways to enhance total pigment content in biomass using stresses such as salinity, light intensity, or nitrogen, or by adding specific precursors, and sometimes through genetic modifications. In the current study, ROS stress has been chosen for pigment production using *Coelastrella thermophila*. ROS stress was used to activate the lutein production pathway in microalgal cells during a two-stage cultivation process.

*Coelastrella thermophila,* a thermotolerant microalga, is known for PUFA and pigment production, which can be increased under certain stress conditions. In general, this strain is reported with 2% lutein, 0.1–0.2% zeaxanthin, with total pigments in its dry biomass. Enhancing pigment production in *Coelastrella thermophila* can be achieved through various strategies, such as optimizing cultivation conditions and media components and manipulating metabolic pathways. This strain can thrive under extreme conditions, such as excess heat, high pH, or intense light, making it a potential producer of microalgal products for commercial purposes. The extremophile nature of *Coelastrella thermophila* offers scope for studying various stress-induced production processes. In the current study, ROS stress has been chosen for pigment production using *Coelastrella thermophila*. Microalgal media supplemented with specific ROS-increasing factors can accumulate carotenoids (Ajitha et al., 2021; León-Vaz et al., 2019; Xi et al., 2021). In the current study, a two-phase microalgal cultivation was carried out to enhance its pigmentation. The first phase, cultivation, was carried out in CSMCRI P2 medium supplemented with a high salt concentration to maximize pigment precursor accumulation, and in the second stage of cultivation, pH was drastically increased to induce ROS stress, resulting in high accumulation of lutein inside the cell. This is the first report of microalgal ROS pigmentation, which produces a single pigment type.

## 2 Materials and methods

### 2.1 Strain

The organism used in this study was *Coelastrella thermophila,* which was isolated from marine brine samples collected from CSMCRI’s Experimental Salt Farm. Fresh *Coelastrella thermophila* P2 used in the present study was taken from the microalgal culture repository of the Integrated Biorefinery laboratory of CSIR-CSMCRI. To ensure the purity of the strain, the centrifuge washing and streak plating techniques were performed to assess its viability. The isolated *Coelastrella thermophila* P2 is being maintained in a petri dish containing Bold Basal Medium (BBM).

### 2.2 Two-stage cultivation of *Coelastrella thermophila*

**Figure 1:**
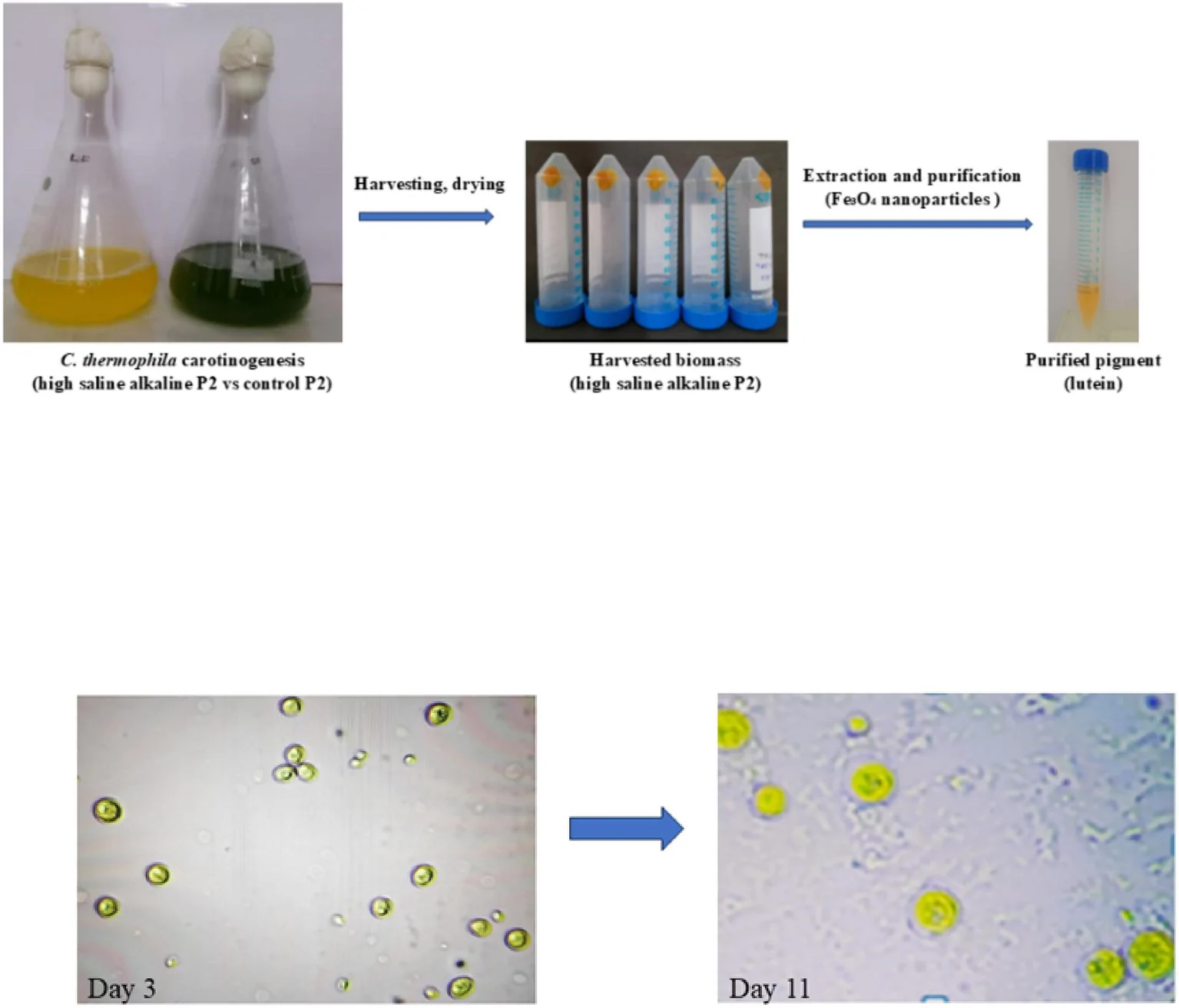
ROS induced pigmentation in *C. thermophila* and extracted pigment, microscopic observation of cellular changes.

Lutein production in *Coelastrella thermophila* was produced in a two-stage cultivation strategy using controlled ROS stress. However, for the first time, we have reported such two stage cultivation wherein at the first stage high density microalgal inoculum was generated and in the second stage, carotenogenesis through stress was provided for obtaining maximum lutein yield (Figure 1). In order to obtain the set of optimized conditions, a combined optimization study was done taking all the stress responsible for carotenogenesis in the primary screening. For microalgal cultivation, initially axenic inoculum of *Coelastrella thermophila* was grown in modified Zarrouk’s medium (CSMCRI P2 medium) consisting of g/l NaHCO_3_ 25; NaNO_3_ 2.5; K_2_HPO_4_ 0.5; K_2_SO_4_ 1.0; NaCl 1.0; CaCl_2_ 0.04; Na_2_EDTA 0.08; MgSO_4_.7H_2_O 0.2; FeSO_4_.7H_2_O 0.01; 1 mL of A5 trace metal solution consisting (%) H_3_BO_3_ 0.286, (NH_4_)6Mo_7_O_24_ 0.002, MnCl_2_.4H_2_O 0.18, CuSO_4_.5H_2_O 0.008, and ZnSO_4_.7H_2_O 0.022, and 1 mL of B6 trace metal solution consisting (%) NH_4_NO_3_ 0.0002296, K_2_Cr_2_(SO_4_)_4_.24H_2_O — 0.000096, NiSO_4_.7H_2_O — 0.004785, Na_2_SO_4_.2H_2_O — 0.001794, Ti(SO_4_)_3_ 0.0040 and Co(NO_3_)2.6H_2_O 0.004948. All the chemicals used were of commercial grade. Modified Zarrouks medium (CSMCRI P2 medium) was used to get maximum growth with maximum biomass as an inoculant. For two stage cultivation for maximum lutein production, the first stage was carried out with high salinity (10 g/L NaCL) and in the second stage, a late log phase wet biomass was transferred into modified CSMCRI P2 media (basic in nature) to cultivate under ROS stress conditions. The first step was to increase the pigment precursor in high salinity and the second was to influence synthesis of the desired pigment, i.e., lutein at high concentration. Overall, first, the P2 media with high salinity was prepared and sterilized at 121 °C for 15 min and once it’s cooled, the selected axenic culture of *Coelastrella thermophila* was inoculated to the media. Thereafter, the inoculated culture flasks were kept in a culture rack under light conditions (16 h light, 8 h dark) and checked for its growth profile (OD, pH, biomass). Once, the culture reaches to log phase, basic pH stress was employed immediately for lutein production and carried for 4 days. After completion of the cultivation, biomass was harvested and dried biomass was used for lutein extraction. All the experiments were done in triplicate.

### 2.3 Medium optimization for improving biomass and lutein productivity

To obtain the optimal media composition for biomass and lutein productivity, the effects of various concentrations of carbon and nitrogen sources were studied. In that regard, 6 experimental runs were conducted varying the concentration of sodium bicarbonate at a concentration of 5 g/L, 10 g/L, 15 g/L, 20 g/L, 25 g/L and 30 g/L was added to Modified Zarrouk’s medium (CSMCRI P2 medium), wherein the rest of the medium constituents were the same as reported previously in Modified Zarrouk’s medium (CSMCRI P2 medium). Simultaneously, 5 experimental runs were conducted varying the concentration of sodium nitrate at a concentration of 1 g/L, 2 g/L, 3 g/L, 4 g/L and 5 g/L was added to Modified Zarrouk’s medium (CSMCRI P2 medium), wherein, the rest of the medium constituents were the same as reported previously in Modified Zarrouk’s medium (CSMCRI P2 medium). Simultaneously, 10 experimental runs were conducted varying the concentration of dipotassium hydrogen phosphate at a concentration of 0.1 g/L, 0.2 g/L, 0.3 g/L, 0.4 g/L, 0.5 g/L, 0.6 g/L, 0.7 g/L, 0.8 g/L, 0.9 g/L and 1 g/L was added to Modified Zarrouks medium (CSMCRI P2 medium), wherein rest all medium constituents were same as reported previously in modified Zarrouk’s medium (CSMCRI P2 medium). Simultaneously, 10 experimental runs were conducted varying the concentration of sodium chloride at a concentration of 1 g/L, 2 g/L, 3 g/L, 4 g/L, 5 g/L, 6 g/L, 7 g/L, 8 g/L, 9 g/L and 10 g/L was added to Modified Zarrouks medium (CSMCRI P2 medium) wherein rest all medium constituents were same as reported previously in modified Zarrouk’s medium (CSMCRI P2 medium) using one factor at a time methodology. The inoculum size was considered 30% (v/v) and the production age 120 h.

### 2.4 Primary screening with different stress for pigmentation

Medium optimization to improve biomass and lutein productivity was carried out using one-factor-at-a-time (OFAT) strategy to obtain optimal upstream conditions, which were thereafter used to improve lutein yield under different ROS stress conditions. During the course of primary screening, the strain of *Coelastrella thermophila* was grown under different ROS stress conditions, aided by high NaCl, H_2_O_2_, high pH (NaOH, KOH), and low pH (HCl), based on previous studies (Supplementary Table 1) under the OFAT strategy (Cirulis et al., 2013; Fal et al., 2022; Tharek et al., 2020; Zuluaga et al., 2017). Visible colour change and the acetone extract of dried biomass were assessed using a UV–Vis spectrophotometer as a primary screening step for maximum lutein production. It was found that extremely high pH (NaOH) and high salinity (NaCl) are both responsible for carotenogenesis of the *C. thermophila* strain. All the experiments were done in triplicate.

### 2.5 Optimization of combined stress effect with salinity and high pH

To determine the exact proportions of pigmenting conditions, a set of variable proportions of salinity and pH was used to cultivate *Coelastrella thermophila*. The concentration of NaOH and NaCl was taken as per Supplementary Table 2. To get an optimized ROS condition, different concentrations of NaOH were employed with variable pH 3 to pH 12, and then the effective pH range 11–16 (4–6% NaOH) was combined and optimized with high salinity (4–7 g/L) using a variable combination of salinity and pH (Supplementary Table 1). After completion of two-stage cultivation (growth phase and carotenoid phase), the cultures were harvested to obtain the desired biomass, and the acetone extracts were analyzed for carotenoids using a spectrophotometric method. All the experiments were done in triplicate.

### 2.6 Growth and pigment estimation

Day-to-day sampling of the cultures was done for regular growth monitoring. For detailed growth estimation, three parameters were observed regularly for each set of experiments, optical density at 680 nm, pH, and dry cell weight. On the other hand, primary quantification and characterization of the pigment were performed by spectrophotometry on an acetone extract of the biomass. Further in this study, a correlation between ROS and average growth rate was examined using the cumulative optical density. All the experiments were done in triplicate.

### 2.7 Chlorophyll and carotenoid estimation

Total pigments were initially extracted from biomass samples obtained through day-to-day sampling using acetone. Further, dried biomass was suspended in acetone, vortexed and sonicated for 2/3 times to allow the chlorophyll and other pigments to be soluble in the solvent, following proper centrifugation to separate out the biomass. The chlorophyll content was estimated by a spectrophotometric assay using the method described by Aminot and Rey (2000). Specifically, optical density was measured at 662 nm, 645 nm, 450 nm, and 470 nm, and the values were calculated using the formula below. Further lutein concentration was determined by measuring optical density at 450 nm and 470 nm. For total carotenoids, optical densities at 450 & 470 nm were used as an indirect measure of lutein concentration, which was further quantified by HPLC from biomass under the optimized conditions. All the experiments were done in triplicate (Equations 1–3).


$$\text{Chlorophyll}\;a,C_{a}=11.75(A622)-2.350(A645)$$ (1)

$$\text{Chlorophyll}\;b,C_{b}=18.61(A645)-3.960(A662)$$ (2)

$$\begin{array}{l} \text{Total carotenoid},C_{x+c}=1000(A470)-2.270\;C_{a}-\\ 81.4\;\mathrm{Ct}/227 \end{array}$$ (3)

### 2.8 Synthesis of Fe₃O₄ nanoparticles

An iron salt solution was prepared by dissolving FeCl₃·6H₂O in deionized water under magnetic stirring to obtain a clear solution. A stoichiometric molar ratio of Fe^3+^:Fe^2+^ = 2:1 was maintained. Furthermore, FeSO₄·7H₂O was prepared separately in deionized water. Both solutions were mixed thoroughly under continuous stirring. Thereafter, the mixed iron salt solution was heated to 70–80 °C under vigorous stirring, and a strong alkaline solution (NaOH or NH₄OH) was added dropwise to the reaction mixture. The mixture was stirred for 30–60 min at 80 °C to ensure complete precipitation and nanoparticle growth. After completion of the reaction, the black precipitate was separated using an external magnetic field and washed repeatedly with deionized water (3–5 times) to remove residual ions and with ethanol (2–3 times) to remove organic impurities. The purified nanoparticles were dried in a vacuum oven at 50–60 °C for 12–24 h. A fine black powder of Fe₃O₄ nanoparticles was obtained and stored in airtight containers for further use.

### 2.9 Extraction of pigment using Fe₃O₄ nanoparticles

Fe₃O₄ nanoparticles were employed as magnetic adsorbents to selectively purify lutein from *Coelastrella thermophila* extracts. Due to their high surface area, magnetic responsiveness, and tunable surface chemistry, Fe₃O₄ nanoparticles offer an efficient and scalable alternative to conventional column chromatography. In this study, the nanoparticles were dispersed in the crude pigment extract under gentle agitation to facilitate the adsorption of fatty acids (the main co-products) onto their surface. Following incubation, magnetic separation enabled the rapid isolation of the FA-bound nanoparticles, effectively removing non-targeted pigments and impurities. Subsequent desorption with tetrahydrofuran (THF) yielded a purified lutein fraction, as confirmed by UV–Vis spectroscopy and HPLC analysis. The process significantly enhanced the purity of lutein while reducing processing time, cost and solvent usage. This method aligns with previous reports on nanoparticle-assisted product separation, demonstrating that Fe₃O₄ can serve as a cost-effective, recyclable, and eco-friendly adsorbent for isolating bioactive compounds (Jafari et al., 2024).

### 2.10 Characterization and quantification of lutein

#### 2.10.1 HPLC analysis

Lutein content in *Coelastrella thermophila* extracts was quantified using high-performance liquid chromatography (HPLC). The analysis was performed on a reverse-phase C18 column with a mobile phase consisting of acetonitrile and methanol in a 40:60 (v/v) ratio. The flow rate was maintained at 0.5 mL/min, and detection was carried out at 445 nm using a UV detector, corresponding to the absorbance maximum of lutein. A lutein standard (≥95% purity) purchased from Sigma-Aldrich was used for quantification. Retention time and peak area were used to identify and quantify lutein in the samples. All measurements were performed in triplicate to ensure reproducibility.

10 mg of the freeze-dried extract was dissolved in 1 mL of methanol to prepare samples. High-Performance Liquid Chromatography (HPLC) measurements were executed with a Waters Alliance 2,695 (Waters)^3^. Samples of 50 μL were injected using an autosampler on a C18-PFP reversed-phase column (150 mm x 4.6 mm, 3 μm particles; Advanced Chromatography Technologies, Aberdeen, Scotland) at a flow rate of 0.80 mL min-1. Chromatographically separation of pigments was done followed by a mobile phase gradient at 0 min of 95% eluent A (70% methanol + 30% 2-Thiobarbituric acid) and 5% of eluent B (100% methanol), at 22 min 5% A and 95% B, at 29 min 5% A and 95% B, at 31 min 95% A and 5% B and at 36 min 95% A and 5% B (Van Heukelem and Thomas, 2001). Identification and quantification of carotenoids were performed by comparison of absorbance spectra and retention times with the reference pigment mix (DHI Laboratory products, Hoersholm, Denmark). The analyses were carried out in triplicate.

#### 2.10.2 NMR characterization

Lutein isolated from *Coelastrella thermophila* extracts was characterized using nuclear magnetic resonance (NMR) spectroscopy to confirm its structural identity. The sample was dissolved in deuterated chloroform (CDCl₃) and analyzed using a JEOL NMR Spectrometer (600 Hz).

#### 2.10.3 UV–VISIBLE (UV–VIS) spectroscopy

The UV–VIS absorption spectrum of lutein extracted from *Coelastrella thermophila* was recorded to assess its characteristic absorbance profile. Initially, lutein estimation was done using a UV–VIS spectrophotometer. The sample was dissolved in acetone and scanned in the range of 300–600 nm. A strong absorbance peak was observed at approximately 445 nm, corresponding to the maximum absorption (*λ* max) of lutein’s conjugated double-bond system. The spectrum was consistent with the known absorbance profile of pure lutein, confirming its presence in the extract.

#### 2.10.4 Characterization of Fe₃O₄ nanoparticles

Fe₃O₄ nanoparticles were characterized using FTIR and XRD. FTIR spectra showed a strong Fe-O stretching vibration near 570 cm^−1^, confirming magnetite formation. XRD patterns exhibited distinct peaks matching the standard magnetite, indicating high crystallinity and phase purity. The size of Fe₃O₄ nanoparticles is 30 nm (Supplementary Figure S8).

#### 2.10.5 Statistical analysis

The mean and standard deviation of the factors involved in the microbial synthesis of lutein in *Coelastrella thermophila* were calculated using the Statistical Package for the Social Sciences (SPSS 25). Also, significance was evaluated using analysis of variance (ANOVA), calculated in SPSS 25. All the experiments were conducted in triplicate.

## 3 Results

### 3.1 Medium optimization for improving biomass and lutein productivity

*Coelastrella thermophila* cultures exhibited varied responses under various concentrations of carbon and nitrogen sources. A biomass yield of 0.8 ± 0.2 g/L was obtained using 25 g/L sodium bicarbonate, with a lutein yield of 2.5 ± 0.3% w/w on the 5th day after inoculation. The inoculum size was considered 30% (v/v) and the production age 120 h. Further, a biomass yield of 0.86 ± 0.2 g/L was obtained using 3 g/L sodium nitrate, with a lutein yield of 3.1 ± 0.3% w/w on the 5th day after inoculation. Growth of *Coelastrella thermophila* at various concentrations of dipotassium hydrogen phosphate: a biomass yield of 0.88 ± 0.2 g/L was obtained using 0.5 g/L dipotassium hydrogen phosphate, with a 3.46 ± 0.3% w/w lutein yield on the 5th day after inoculation. Further, a biomass yield of 0.89 ± 0.3 g/L was obtained using 7 g/L sodium chloride, with a lutein yield of 4.21% w/w on the 5^th^ day after inoculation.

### 3.2 Screening with different stress for pigmentation and growth curve

**Figure 2:**
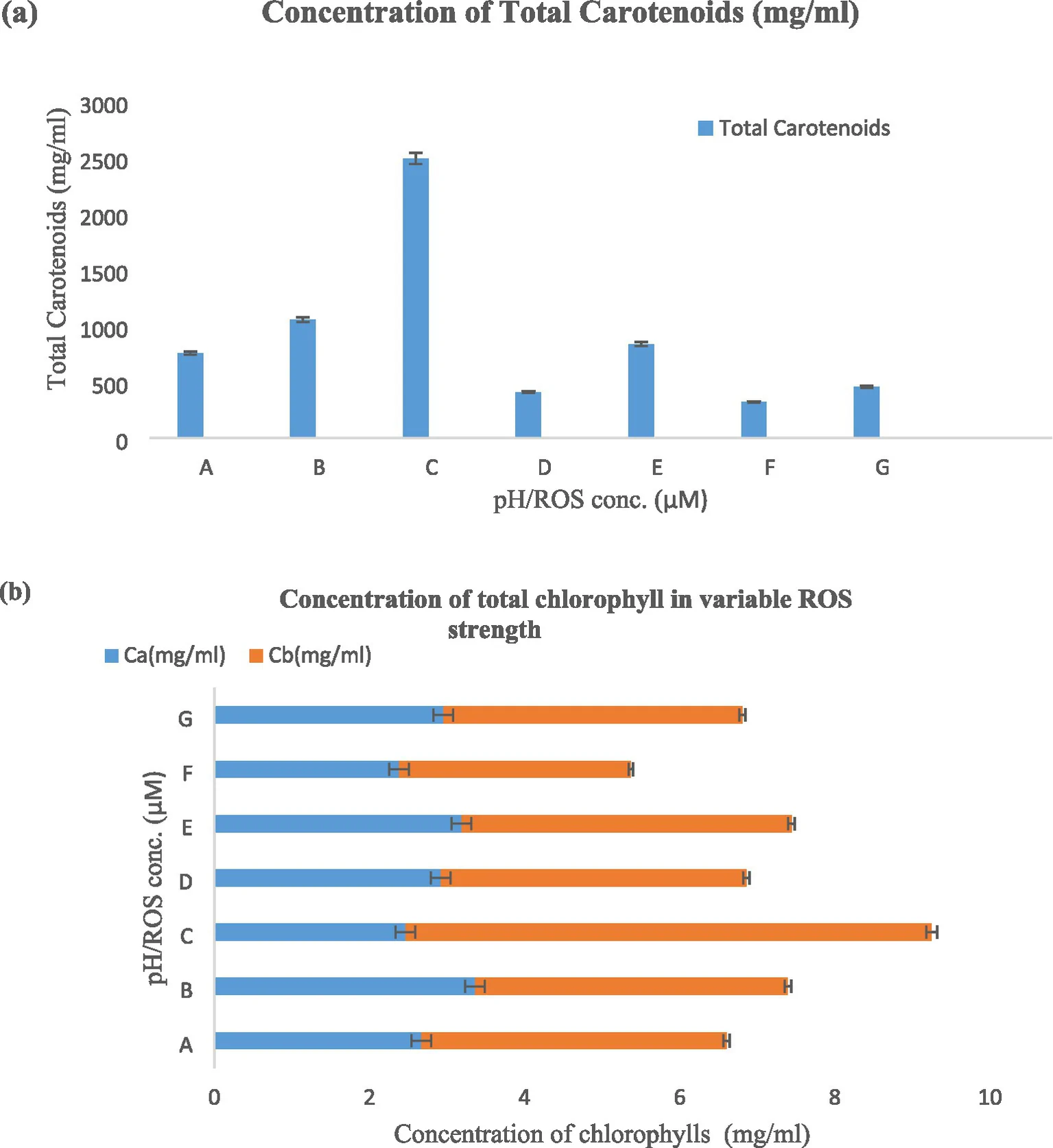
Effect of basic pH-induced oxidative stress on pigment accumulation in *C. thermophila*. Experimental conditions were: A = 14.0 pH, B = 14.5 pH, C = 14.8 pH, D = 15.0 pH, E = 15.5 pH, *F* = 15.8 pH, G = 16.0 pH, and Control = 7.0 pH. Carotenoid and chlorophyll concentrations were determined spectrophotometrically. **(a)** Concentration of Total Carotenoids (mg/ml). **(b)** Concentration of total chlorophyllin variable ROS strength.

*Coelastrella thermophila* cultures exhibited varied responses under different abiotic stress conditions (Supplementary Table 1). In the present case, two-stage cultivation was monitored by measuring optical density at 680 nm for routine growth estimation, and carotenogenesis at the second stage was monitored by measuring the optical density at 450 nm of the acetone extract of biomass (Figure 2). Under salinity stress induced by NaCl, there was no visible colour change but an increase in optical density at 450 nm, indicating minimal effect on pigment composition, which aligns with previous findings showing that moderate salinity increase can result carotenoid biosynthesis in certain microalgal strains. Exposure to oxidative stress with hydrogen peroxide (H₂O₂) showed no observable change, suggesting tolerance or insufficient ROS induction, consistent with studies in which low concentrations of H₂O₂ did not significantly affect microalgal morphology or pigment content (Zhou et al., 2018). Alkaline stress from high pH caused noticeable colour shifts, with cultures treated with KOH appearing greenish-yellow and those exposed to NaOH turning yellowish-orange. Such pH-induced pigment alterations have been previously reported and are often linked to changes in chlorophyll degradation and carotenoid accumulation under ROS stress as a cellular defence mechanism. In contrast, acidification of the medium to pH 5.5 with HCl completely inhibited growth, highlighting the microalgae’s intolerance to low pH and corroborating earlier reports of suppressed photosynthetic activity and impaired cell division at acidic pH levels.

### 3.3 Total carotenoids and chlorophyll changes under different ROS concentrations

**Figure 3:**
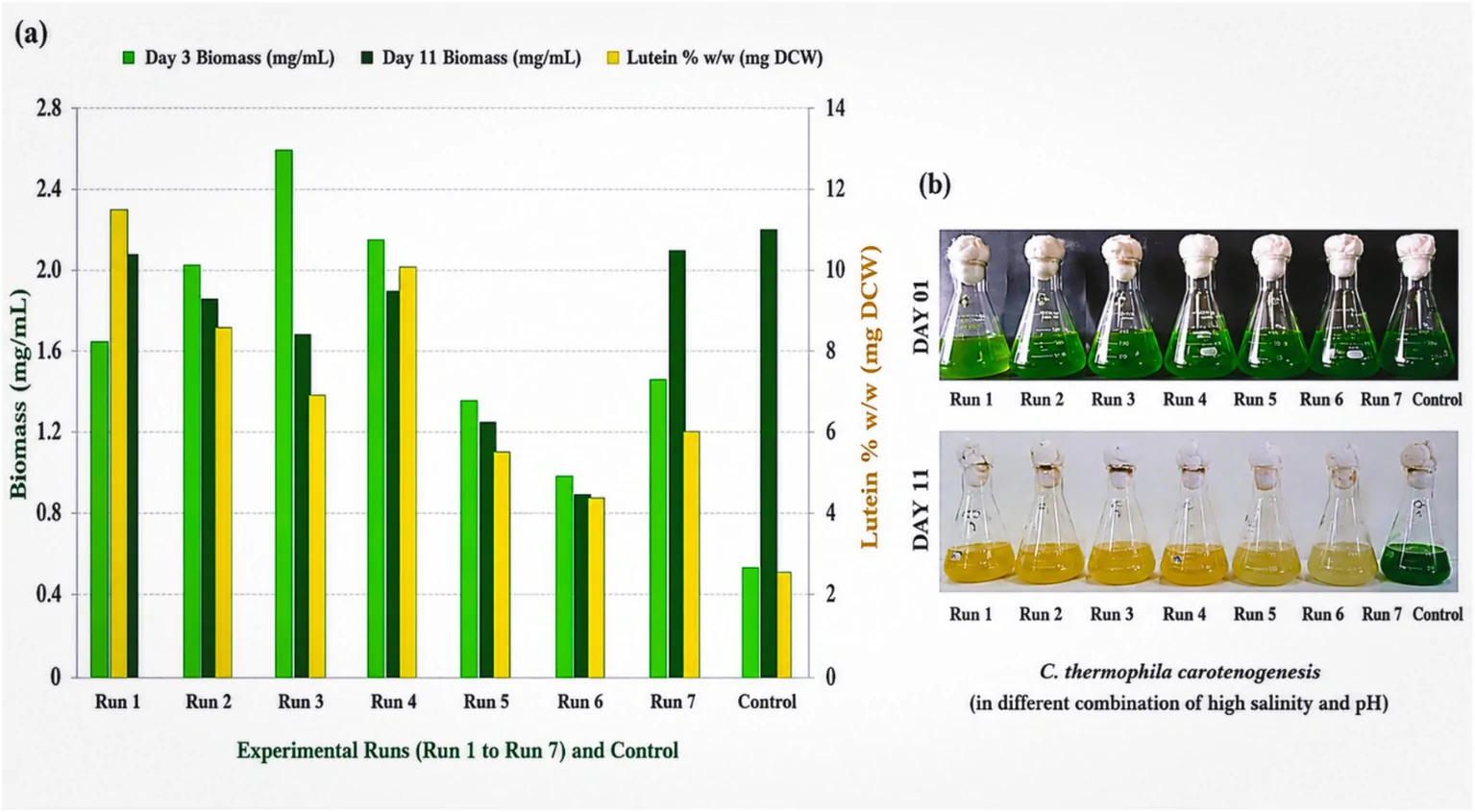
**(a)** Effect of various concentration of NaCl (1–8%) on biomass and lutein (% w/w w.r.t. CDW). Experimental conditions were: Run 1 = 8.24% NaCl, 13.0 pH; Run 2 = 7% NaCl, 13.5 pH, Run 3 = 7% NaCl, 14 pH, Run 4 = 4% NaCl, 14.5 pH, Run 5 = 4% NaCl, 15 pH, Run 6 = 4% NaCl, 15.5 pH, Run 7 = 1% NaCl, 16.0; pH, and Control = 1% NaCl, 7.0 pH. **(b)** Visual confirmation of lutein production in culture varying concentration of NaCl (1–8%). Experimental conditions were: Run 1 = 8.24% NaCl, 13.0 pH; Run 2 = 7% NaCl, 13.5 pH, Run 3 = 7% NaCl, 14 pH, Run 4 = 4% NaCl, 14.5 pH, Run 5 = 4% NaCl, 15 pH, Run 6 = 4% NaCl, 15.5 pH, Run 7 = 1% NaCl, 16.0; pH, and Control = 1% NaCl, 7.0 pH.

The influence of ROS generated under alkaline stress was evaluated by analyzing pigment profiles across a range of pH levels at high salinity. At elevated pH values (Cristobal et al., 2024; Eilers et al., 2016; Fal et al., 2022), corresponding to high ROS conditions (graph 2/C), a marked enhancement in total carotenoid accumulation was observed, while chlorophyll levels remained moderate. In contrast, cultures maintained at pH 9 (Figure 3) exhibited the highest chlorophyll content but the lowest lutein-to-total carotenoid ratio, indicating minimal production of protective carotenoids. These results also suggest that increasing salinity and the associated oxidative stress from pH act as a physiological trigger for carotenoid biosynthesis, likely as a defence mechanism against photo-oxidative damage. This inverse relationship between carotenoid and chlorophyll levels aligns with previous findings that xanthophylls, including lutein, are upregulated under stress conditions to safeguard the photosynthetic apparatus. Overall, these findings reinforce the hypothesis that alkaline-induced oxidative stress stimulates the carotenoid pathway while compromising chlorophyll accumulation.

### 3.4 Optimization of combined stress effect with salinity and high pH

**Figure 4:**
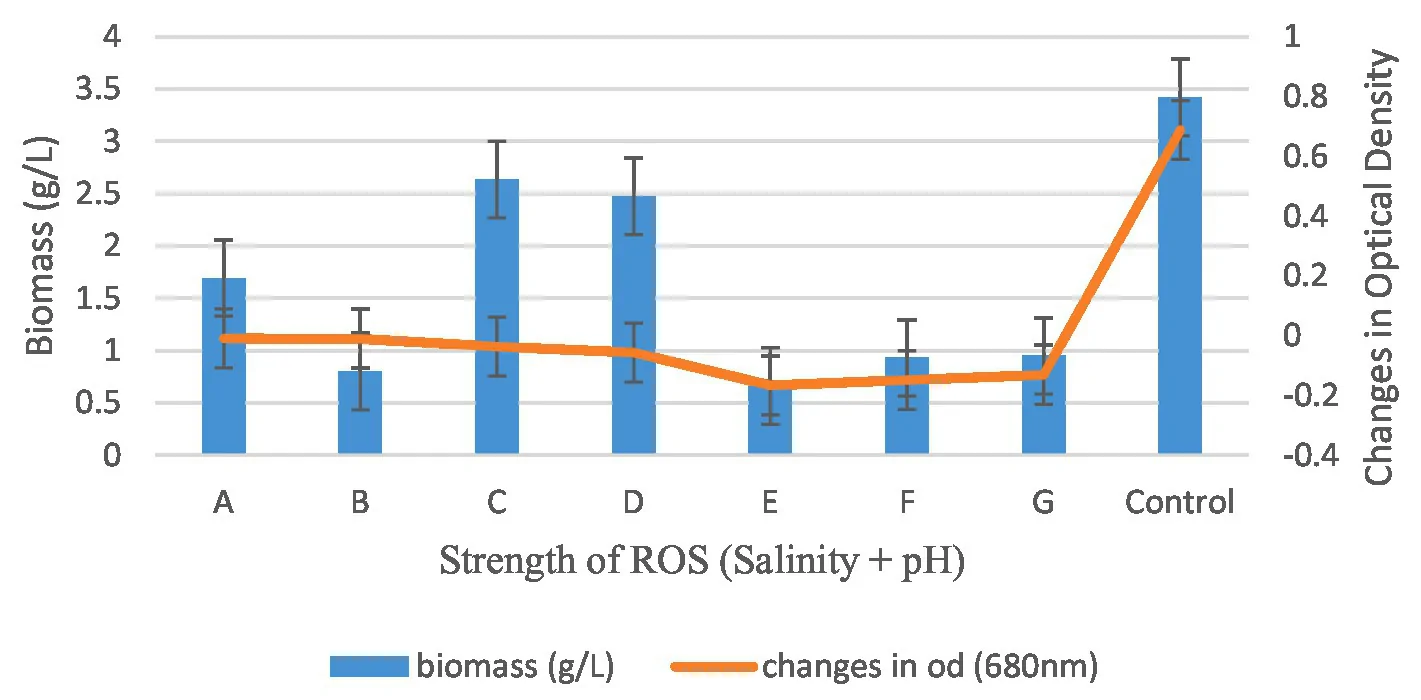
Growth response of *C. thermophila* under different levels of basic pH-induced oxidative stress. Optical density was measured at 680 nm [OD (680 nm)]. Experimental conditions were: A = 14.0 pH, B = 14.5 pH, C = 14.8 pH, D = 15.0 pH, E = 15.5 pH, F = 15.8 pH, G = 16.0 pH, and Control = 7.0 pH.

A combined stress of salinity & pH (ROS) produces pigment in *Coelastrella thermophila*. However, to optimize the lutein process under these conditions, it is necessary to determine an optimal combination of pH and salinity without cell degradation. In Figure 4, run 3 has increased biomass as well as increased pigmentation - the stress proportion which *Coelastrella thermophila* can tolerate through carotenogenesis (Supplementary Table S2). Other runs, also showed yellow biomass but decreased biomass (i.e., cell reduction) due to extreme ROS (Supplementary Tables S1, S2).

### 3.5 Biomass kinetics under ROS-induced alkaline stress

Spectrophotometric analysis revealed distinct shifts in pigment composition and biomass productivity in response to ROS generated under varying alkaline conditions. Optical density at 680 nm (OD₆₈₀), a measure for chlorophyll content and photosynthetic activity, declined under elevated pH, reflecting stress-induced suppression of chlorophyll synthesis and possible structural damage to the photosynthetic apparatus. In contrast, Optical density at 450 nm (OD₄₅₀), indicative of lutein accumulation, showed a marked increase under high ROS conditions, consistent with upregulation of xanthophyll-cycle pigments as a photoprotective response. Biomass productivity followed a biphasic trend under near-neutral to moderately alkaline conditions, with biomass increasing, suggesting optimal growth; however, the sharp reduction in biomass is likely due to uncontrolled oxidative stress-induced cell lysis, leading to metabolic arrest. Among the experimental treatments (Samples A–G, Figure 4), samples C and D, corresponding to pH 11–12, emerged as optimal, demonstrating enhanced OD₄₅₀ and moderate biomass retention, signifying effective lutein induction without extensive compromise to cellular integrity. Microalgal samples from C & D had average biomass compared to the control in Figure 4, while a decrease in cumulative OD from day 2 to day 13 confirms the best-optimized condition under ROS-carotenogenesis. These results underscore the physiological trade-off between pigment-based photoprotection and growth under oxidative stress, supporting earlier hypotheses that high pH conditions can selectively promote carotenoid biosynthesis at the expense of chlorophyll stability and biomass yield.

*p* < 0.05 on ANOVA of the microalgal growth. ROS-induced alkaline stress in all cases signifies the productivity boost over control.

## 4 Characterization and quantification of pigment

### 4.1 UV spectra

The UV–Visible spectrophotometric analysis of the extracted pigment revealed distinct absorption features consistent with lutein. The purified sample displayed a characteristic triple-peak pattern in the visible range, with major absorption maxima at approximately **445 nm and 472 nm**, and a shoulder near **422 nm**, which closely aligns with the known spectral fingerprint of lutein reported in previous studies (Britton, 1995; Rodriguez-Amaya, 2001). These peaks are attributed to the conjugated double-bond system in the lutein molecule, which is responsible for its light-absorbing properties in the blue region of the spectrum (Supplementary Figure S2).

### 4.2 Lutein characterization by HPLC method

Qualitative and quantitative analyses of lutein content in *Coellastrella thermophila* cultivated under different ROS conditions were performed using HPLC and summarized in Table 1.

**Table 1:** Concentration of lutein content identified in HPLC in *Coellastrella thermophila* cultivated in different ROS conditions.

Run	NaCl %	pH	Total biomass (Day 11) (mg/ml)	Concentration of lutein (mg/ml)
1	8.24	13.0	1.03 ± 0.01	0.104 ± 0.03
2	7	13.5	0.9 ± 0.1	0.05 ± 0.01
3	7	14.0	0.73 ± 0.2	0.0015 ± 0.02
4	4	14.5	0.59 ± 0.1	0.046 ± 0.01
5	4	15.0	0.1	0.0039 ± 0.03
6	4	15.5	0.75 ± 0.02	0.024 ± 0.01
7	1	16.0	0.58 ± 0.04	0.0035 ± 0.005
Control	1	7.0	1.07 ± 0.1	0.024 ± 0.01

The results demonstrated that on the 11th day of cultivation, maximum biomass of 1.03 ± 0.01 mg/mL containing 0.104 ± 0.03 mg/mL lutein was obtained in *Coellastrella thermophila* in modified Zarrouk’s medium (P2 medium) containing 8.24% sodium chloride and 4% sodium hydroxide w/v (pH 14). Also, the HPLC results showed a single lutein peak (Supplementary Figure S1).

### 4.3 NMR

**Figure 5:**
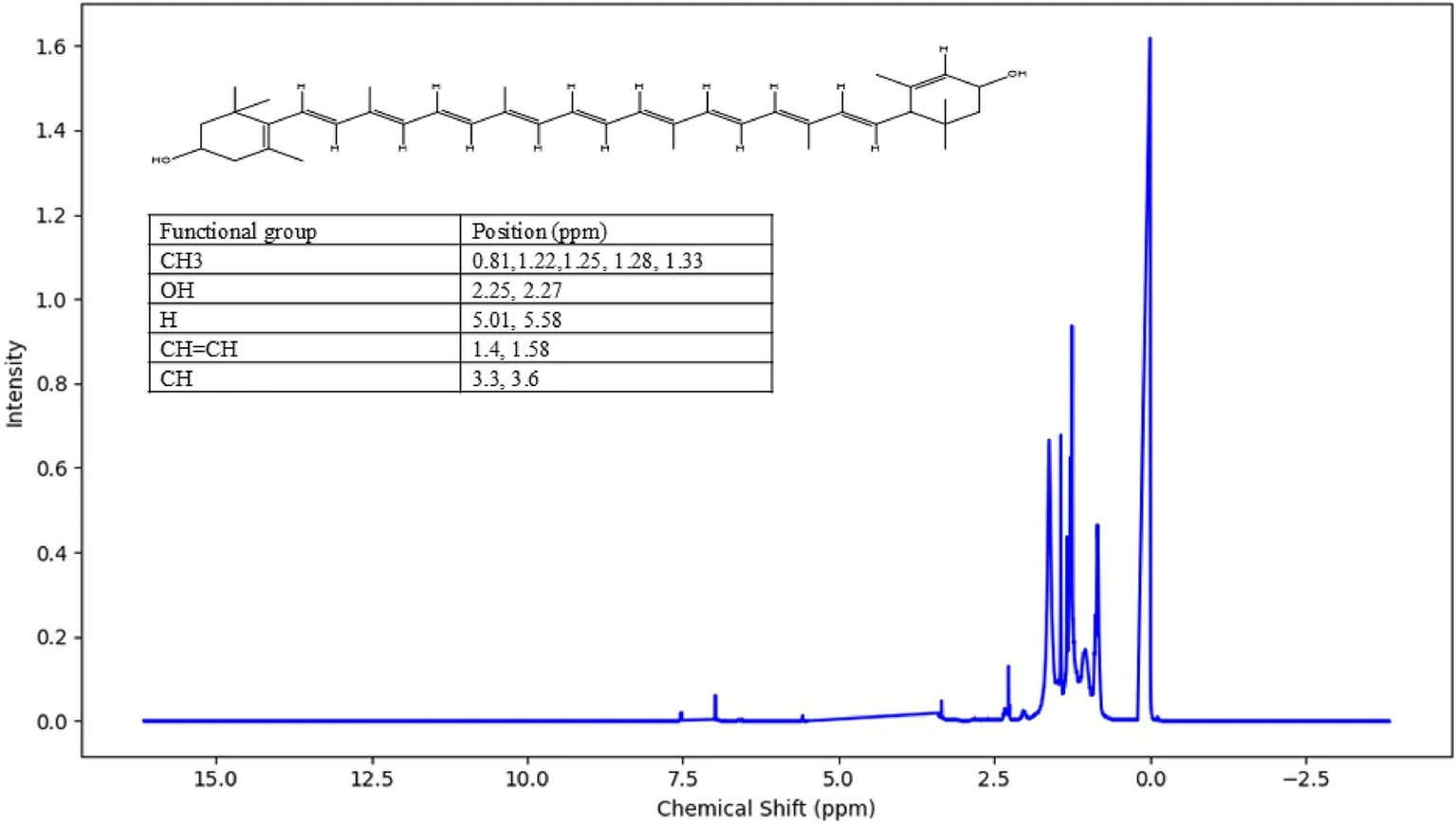
NMR spectrum of purified pigment extract.

Interpretation of 1H NMR showed the presence of the functional group of Lutein, while detection of the intermediate CH- group was rarely found. Further shift at 5.5 and 1.8 confirms the presence of conjugation at ‘H-CH=CH-H’. Similarly, the presence of the ‘OH-’ group was confirmed from the peak at 3.3 to 3.6. Other significant peaks were found at 0.81, 1.22, 1.25, 1.28, and 1.33 for different types of CH_3_ groups; 2.25, 2.27 for OH groups; 5.01, 5.58 for terminal H; 1.4, 1.58 for CH=CH; and 3.3, 3.6 for intermediate CH groups (Figure 5).

## 5 Discussion

The newly isolated *Coelastrella thermophila* was found to produce high levels of carotenoids under extreme salinity and high pH conditions, reflecting its self-defence mechanism against ROS. Coelastrella *thermophila* is a thermophilic green microalga recognized for its robust industrial potential, particularly due to its ability to thrive in extreme environments. It is highly valued for producing biofuel, carotenoids, and other valuable compounds in a single, cost-effective growth cycle. The CSMCRI’s isolated *Coelastrella thermophila* grows at high temperatures (> 35 °C), making it suitable for industrial processes, and it grows easily and cost-effectively in open raceway ponds and at the industrial site that generates heat, thereby reducing cooling costs.

Lutein biosynthesis in microalgae primarily proceeds through the plastidial methylerythritol phosphate (MEP) pathway, which is the dominant route for isoprenoid formation in most photosynthetic microalgae (Lou et al., 2021). This pathway begins with the condensation of pyruvate and glyceraldehyde-3-phosphate (G3P) to form 1-deoxy-D-xylulose-5-phosphate (DXP), which is then converted into 2-C-methyl-D-erythritol 4-phosphate (MEP) via DXP reductoisomerase (Liang et al., 2016). Through several enzymatic steps, MEP is converted into isopentenyl pyrophosphate (IPP) and dimethylallyl pyrophosphate (DMAPP), the five-carbon precursors of carotenoids (Cordero et al., 2011). These intermediates are condensed to form geranylgeranyl pyrophosphate (GGPP), which is converted to phytoene by phytoene synthase (PSY), initiating the carotenoid-specific branch of the pathway. Phytoene undergoes desaturation and isomerization through enzymes such as phytoene desaturase (PDS), *ζ*-carotene desaturase (ZDS), and carotenoid isomerase (CRTISO), resulting in the formation of all-trans-lycopene (Fraser and Bramley, 2004; Ralley et al., 2004). Lycopene is then cyclized by lycopene *β*-cyclase (LCYB) and lycopene *ε*-cyclase (LCYE) to yield *α*-carotene, the direct precursor of lutein (Li et al., 2020). Subsequent hydroxylation of α-carotene by β-ring hydroxylase (CHYB) and ε-ring hydroxylase (CHYE) leads to the production of lutein (Eilers et al., 2016). The expression of LCYE and CHYE is particularly critical in directing metabolic flux toward lutein instead of other xanthophylls like zeaxanthin. Environmental cues such as light intensity, carbon source, and nutrient availability have been shown to regulate the transcriptional and post-translational levels of these enzymes, influencing lutein accumulation under autotrophic, heterotrophic, and mixotrophic conditions (Liang et al., 2016; Qin et al., 2024). Therefore, a detailed understanding of the MEP pathway and its regulatory network is essential for metabolic engineering and cultivation optimization to maximize lutein yield in microalgae. Under conditions of high light intensity, the reaction centers cannot process all the energy they receive. Consequently, triplet state chlorophylls are formed, which in turn react with oxygen, forming Reactive Oxygen Species (ROS) (Larkum et al., 2020). These ROS can damage lipids, proteins, and nucleic acids, all of which are essential for the photosynthetic apparatus and, more broadly, for cell integrity. To prevent this damage, lutein can absorb energy from triplet-state chlorophyll and singlet oxygen, forming a triplet-state lutein that returns to the stable ground state by safely releasing the excess energy as heat (Sun et al., 2018). Moreover, lutein can scavenge ROS that would otherwise be formed (Jahns and Holzwarth, 2012).

The relationship between ROS (Reactive Oxygen Species) intensity and lutein production is a dynamic, **biphasic “stress-response” mechanism**. Lutein is a key component of the antioxidant defence system: moderate-to-high ROS levels induce its production as a protective mechanism, whereas extreme stress can lead to its degradation. Moderate ROS levels act as signals that trigger the upregulation of lutein biosynthesis, particularly in microalgal species facing abiotic stress involving high light, salinity, or high temperature. The highest lutein productivity in microalgae often occurs at light intensities that create moderate stress, rather than at lower light levels (growth stage) or extremely high light levels (severe damage stage), resulting in ROS intensity that triggers lutein biosynthesis. The study by Vadrale et al. (2023) also aimed to determine the correlation between changes in ROS levels and lutein content in microalgal cells. Vadrale et al. (2023) reported that on day 8th at stage II, ROS was higher in the control and decreased with increasing lutein content. The study by Vadrale et al. (2023) also showed that with increasing lutein content, ROS levels gradually decreased. Induced oxidative stress in microalgae significantly influences the production of antioxidant compounds such as lutein. When microalgae are exposed to non-optimal pH conditions, either acidic or alkaline, it can disrupt cellular homeostasis and lead to the excessive generation of reactive oxygen species (ROS). This oxidative stress triggers the microalgae’s defence mechanisms, including the upregulation of carotenoid biosynthesis pathways, to mitigate ROS damage.

Lutein is linked to photosystem proteins and is involved in the light-harvesting complex, fulfilling three primary functions: (i) it aids in the proper folding of photosystem II proteins; (ii) it absorbs blue-green light to enhance photosynthetic efficiency in low light conditions, transferring the absorbed energy to chlorophyll; and (iii) it serves as an antioxidant. Under conditions of elevated light intensity, the reaction centers are unable to assimilate all the energy they receive. As a result, triplet-state chlorophylls are generated, which subsequently interact with oxygen to produce Reactive Oxygen Species (ROS). These reactive oxygen species can damage lipids, proteins, and nucleic acids, which are vital to the photosynthetic apparatus and, more generally, to cellular integrity. Lutein can mitigate damage by absorbing energy from triplet-state chlorophyll and singlet oxygen, resulting in the formation of triplet-state lutein, which subsequently returns to a stable ground state by dissipating excess energy as heat (Camarena-Bernard and Pozzobon, 2024).

Diverse microalgal species, including *Chlorella* and *Scenedesmus*, are exposed to elevated pH levels (occasionally exceeding pH 11) in cultivation media due to the incorporation of bicarbonates and nitrogen sources (e.g., nitrates), which liberate hydroxide ions (OH^−^) and deplete bicarbonate. The alkaline environment induces cellular stress, leading to an elevation of Reactive Oxygen Species (ROS) within cells, including H₂O₂, radicals, and singlet oxygen. In response to oxidative damage, microalgae augment the antioxidant defence systems of their photosynthetic apparatus, leading to upregulation of carotenogenic pathways that specifically promote lutein production to neutralize reactive oxygen species and stabilize chloroplast membranes. Numerous reports have indicated that stress conditions, including pH and nutrient stress, upregulate genes associated with carotenoid biosynthesis, such as phytoene desaturase (PDS) and lycopene cyclase (LCYe), thereby augmenting metabolic flux toward lutein biosynthesis. Manipulating pH levels in cultivation systems can be a strategic approach to boosting lutein yield in microalgal biomass for nutraceutical and pharmaceutical applications. Under stress conditions, the lutein biosynthesis pathway in microalgae is significantly upregulated compared to its activity under normal physiological states. Under stress conditions, lutein is synthesized via the methylerythritol phosphate (MEP) pathway, beginning with pyruvate and glyceraldehyde-3-phosphate and leading to the production of isoprenoid intermediates. Enzymes such as phytoene synthase (PSY), phytoene desaturase (PDS), and zeta-carotene desaturase (ZDS) sequentially convert these intermediates into lycopene and then to *α*-carotene, which is hydroxylated to form lutein. However, under oxidative stress induced by agents like hydrogen peroxide (H₂O₂) and sodium hypochlorite (NaClO), *Chlorella protothecoides* exhibits elevated levels of reactive oxygen species (ROS), which serve as signalling molecules to activate carotenoid biosynthesis genes, especially PSY and downstream enzymes, enhancing lutein accumulation as a cellular defense mechanism (Fu et al., 2014; Kehrer, 2000; Wei et al., 2008).

Heterotrophic and mixotrophic cultivation modes have demonstrated distinct impacts on lutein production in microalgae, with numerous studies highlighting the superiority of mixotrophic conditions in enhancing both biomass and lutein yield. Heterotrophic cultivation, relying exclusively on organic carbon sources in darkness, promotes rapid biomass accumulation but often leads to reduced lutein content due to suppressed photosynthetic activity. For instance, Cristobal et al. (2024) reported that *Scenedesmus almeriensis* achieved a biomass productivity of 1.4 g/L/day under heterotrophic conditions, but the lutein content was comparatively lower (~3.5 mg/g dry weight), underscoring pigment synthesis limitation in the absence of light. Conversely, mixotrophic cultivation combines the benefits of autotrophic photosynthesis and heterotrophic carbon assimilation, fostering enhanced lutein biosynthesis. Bentahar and Deschênes (2022) used a multinutrient kinetic model to optimize the mixotrophic culture of microalgae, achieving lutein yields up to 12 mg/L and biomass productivity exceeding 1.7 g/L/day by fine-tuning nutrient ratios and substrate feeding strategies. Further, the use of crude glycerol as a cost-effective carbon source in airlift photobioreactors was explored by Rajendran et al. (2020), achieving biomass productivity of 1.8 g/L/day and lutein content of 7.4 mg/g, highlighting sustainable substrate utilization in mixotrophy. In direct comparisons, Yen et al. (2011) found that *Scenedesmus* sp. cultivated mixotrophically yielded 6.5 mg/g dry weight of lutein, surpassing autotrophic (3.8 mg/g) and heterotrophic (2.9 mg/g) cultures, confirming enhanced pigment production efficiency under combined metabolic modes. Liaqat et al. (2023) further reviewed multiple microalgal species and concluded that mixotrophic cultivation consistently increases carotenoid yields, including lutein, by 30–70% relative to other growth modes, which they attributed to increased energy availability and metabolic flexibility. Collectively, these studies substantiate that mixotrophic cultivation optimizes lutein synthesis by integrating light-dependent biosynthesis with organic substrate metabolism, enabling both high biomass productivity and pigment accumulation, thus offering an efficient strategy for scalable lutein production. Furthermore, metabolic engineering via overexpression of the PSY, LCYe, and CYP97 genes can play a pivotal role in enhancing lutein production, thereby making the process more sustainable and scalable. Conventionally, carotenoid/lutein extraction is performed using organic solvents such as chloroform, hexane, isopropanol, methylene chloride, or diethyl ether (Kultys and Kurek, 2022). Synthetic methods for lutein production have been developed; however, the cost of producing synthetic lutein is incompatible with that of isolating it from plant biomass (Fernández-Sevilla et al., 2010). Further, the organic solvents used for lutein extraction, such as hexane, which are sourced mainly from non-renewable resources, are flammable, volatile, and toxic, and contribute to environmental pollution (Sicaire et al., 2016). However, petrochemical-derived solvents, such as hexane, are now strictly regulated by European Directives and the Registration, Evaluation, Authorization, and Restriction of Chemicals (REACH), posing a major concern for their industrial use. However, Kashyap et al. (2022) have utilized 2-methyltetrahydrofuran as a green solvent for lutein extraction from marigold flowers, with a 1.39% yield (Chemat et al., 2019; Kultys and Kurek, 2022; Kashyap et al., 2022).

Iron oxides are widely distributed in nature and can be easily synthesized in the laboratory (Mohajerani et al., 2019; Ali et al., 2016; Andra et al., 2019; Radoń et al., 2019; Schwaminger et al., 2017). There are 16 iron oxides, one of the most common being magnetite (Fe3O4), and they can be present in their two aggregation states, as Fe^+2^ and Fe^+3^ forming oxides (Bhole et al., 2023; Giraldo et al., 2013; Fato et al., 2019; Mahdavi et al., 2012). Most iron oxides are crystalline. Magnetite is an iron oxide noted for its magnetic properties. Magnetite nanoparticles exhibit a large surface area that allows them to be functionalized to make biocompatible products (porous surface) (Radoń et al., 2019; Schwaminger et al., 2017; Bhole et al., 2023; Giraldo et al., 2013; Fato et al., 2019; Mahdavi et al., 2012; Ali et al., 2017; Pirsaheb and Moradi, 2021). This characteristic makes them especially suitable for a wide range of applications in diverse areas such as biotechnology, environmental, medical, pharmaceutical, catalysts, pigments, gas sensors, optical and electromagnetic devices, decontaminants, and wastewater treatment for dye removal (Giraldo et al., 2013; Fato et al., 2019; Mahdavi et al., 2012; Ali et al., 2017; Pirsaheb and Moradi, 2021).

Similarly, in *Chlorella sorokiniana* FZU60, a two-stage cultivation strategy involving mixotrophy followed by photoautotrophy acts as a controlled stress environment. Transcriptomic data reported by Ma et al. (2023) revealed significant upregulation of carotenoid biosynthetic genes, correlating with enhanced lutein content in the cells. Overall, under stress conditions, the lutein biosynthesis pathway becomes a tightly regulated, upshifted process that serves both protective and functional roles in microalgal physiology. Lutein accumulation is enhanced under high-light, nitrogen-starvation, and other stress conditions. Conventional purification methods for lutein are based on extracting it from marigolds. The basic steps for the extraction and purification of lutein involve extracting crude pigment from dry biomass using organic solvents such as hexane, ethanol, acetone, or petroleum ether. However, Soxhlet extraction is sometimes used for continuous solvent extraction, followed by saponification (alkaline hydrolysis), because lutein in plants often exists as fatty acid esters. Further, treatment with an alkali (commonly KOH in methanol or ethanol) removes fatty acids and chlorophyll impurities, converting lutein esters into free lutein, followed by washing with water and separating the organic and aqueous phases to remove the alkali and unwanted compounds. Finally, the excess solvents are removed by evaporation to obtain crude lutein. The crude lutein is then subjected to Column chromatography, followed lutein may be obtained as lutein crystals or powder.by recrystallization using solvents such as ethanol or hexane mixtures, and the purified lutein is dried under vacuum or in an inert atmosphere to avoid oxidation.

On the other hand, the extraction and purification of lutein using Fe₃O₄ nanoparticles is a single-step process in which lutein is purified via adsorption by the prepared nanoparticles. However, if the lutein is in the form of lutein esters, saponification can be done to convert the lutein from lutein esters to free lutein.

Various reports indicate that optimized nanoparticle extraction systems can achieve lutein recovery rates of 85–95%, compared with conventional solvent extraction methods, which typically yield 60–75% under comparable conditions. This differential arises from several mechanistic factors. The high surface area of the nanoparticles ensures that even trace concentrations of lutein in dilute extracts can be captured efficiently, rather than being lost in the raffinate or discarded wash fractions. The selectivity conferred by surface functionalization also reduces lutein loss via nonspecific co-adsorption pathways that complicate conventional purification.

Furthermore, the magnetic separation step eliminates the need for centrifugation or vacuum filtration - unit operations that are known to cause mechanical shear and thermal stress on delicate carotenoid molecules. By replacing these harsh physical processes with a gentle magnetic collection mechanism, the nanoparticle approach preserves lutein’s structural integrity, maintaining higher biological activity and pigment purity in the final product. This is particularly important for pharmaceutical-grade applications where isomeric composition and oxidative degradation are closely monitored.

Using optimized nanoparticle extraction systems, such as those using Fe₃O₄ nanoparticles, is more reproducible and easier to handle than conventional purification methods. The estimated cost of production using optimized nanoparticle-assisted extraction at pilot scale is USD 180–450 per kg; however, using conventional solvent extraction costs USD 250–600 per kg of purified lutein, and USD 400–900 per kg using supercritical CO₂ extraction.

Recovery of lutein using Fe₃O₄ (magnetite) nanoparticles is a promising nanotechnology-based purification method; however, several technical, economic, and operational challenges still limit its large-scale application. During nanoparticle processing, catalytic metal surfaces may accelerate oxidation, prolonged stirring can increase degradation, and reactive oxygen species may form, resulting in reduced recovery efficiency. Further, during scale-up, poor selectivity can lead to incomplete purification, lutein loss during magnetic separation and contamination in the final product. Another important limitation of the process is that residual nanoparticles may remain in the lutein product after purification.

## 6 Conclusion

The newly isolated *Coelastrella thermophila* was found to produce high levels of carotenoids under extreme salinity and high pH conditions, reflecting its self-defence mechanism against ROS. Excessive pH via NaOH accumulation of ROS via the Fenton reaction, which facilitates the reduction of pigment backbones by the free radical OH inside the cell, is expressed as high concentrations of xanthophylls in algal cells. Lutein is an oxidized derivative of the common carotene backbone, and its formation requires a more oxygen-rich environment, which can be provided by external oxidative stress. Interestingly, the algal species *Coelastrella thermophila* exhibited a novel mechanism for producing a single pigment under optimized ROS stress, which was identified as lutein.

## Data Availability

The original contributions presented in the study are included in the article/Supplementary material, further inquiries can be directed to the corresponding authors.

## Footnotes

1
https://www.grandviewresearch.com/industry-analysis/global-astaxanthin-market

2
https://www.grandviewresearch.com/industry-analysis/lutein-market-report

3
http://www.waters.com
