# Supplementary material for: Sustainable process for increased lutein production in green microalga Coelastrella thermophila
Source: Front Microbiol. 2026 Aug 10;17:1833061. doi: 10.3389/fmicb.2026.1833061 (PMC13500628; doi:10.3389/fmicb.2026.1833061)
Supplement: Supplementary file 1 [file Data_Sheet_1.pdf]

Supplementary Information

Sustainable Process for increased lutein production in green microalga *Coelastrella thermophila*

Sudipto Adhikary[1,2], Sejal Dangolia[1], Mirnalini Juyal[2,3], Rushi Joshi[1], Debarati Paul[1], Debashish Ghosh[2,3], Shreyansh Jain[4,5]* and Sourish Bhattacharya[1,2]*

[1]Applied Phycology and Biotechnology Division, CSIR-Central Salt and Marine Chemicals Research Institute, Bhavnagar, India.

[2]Academy of Scientific and Innovative Research (AcSIR), CSIR-HRDC
Campus, Ghaziabad, Uttar Pradesh, India.

[3]Material Resource Efficiency Division, CSIR-Indian Institute of Petroleum, Dehradun, Uttarakhand, India.

[4]Intrinsic Foundries Inc., 56 Broad Street, Boston, MA 02109, United States.

[5]Intrinsic Foundries, IIOT Innovation Private Limited, 2nd Floor, Vinayaka Tower, Hazaribagh 825301, Jharkhand, India.

Materials and Methods

Thin Layer Chromatography (TLC) Analysis of Lutein

Methodology

Thin layer chromatography (TLC) was performed to separate and identify lutein present in the extracted samples. Silica gel 60 F254 precoated aluminum TLC plates were used as the stationary phase.

Preparation of Sample

The extracted lutein sample was dissolved in a small volume of acetone and filtered through Whatman No. 1 filter paper to remove insoluble particles. A standard lutein solution was prepared similarly for comparison.

Preparation of Mobile Phase

The mobile phase consisted of hexane and acetone in the ratio of 70:30 (v/v). The solvent mixture was prepared freshly and transferred into a TLC developing chamber. The chamber was saturated with solvent vapor for 15–20 min before chromatography.

Spotting Procedure

A baseline was drawn approximately 1.5 cm above the lower edge of the TLC plate using a pencil. Using a capillary tube, 5–10 µL of the sample extract and standard lutein solution were separately spotted onto the marked positions on the plate. The spots were allowed to dry at room temperature.

Chromatographic Development

The spotted TLC plate was carefully placed inside the developing chamber containing the mobile phase, ensuring that the sample spots remained above the solvent level. The plate was developed until the solvent front migrated approximately 80% of the plate length.

The plate was then removed, and the solvent front was immediately marked with a pencil. The developed plate was air-dried under dark conditions.

Visualization and Identification

Separated bands were visualized under visible light and UV light (365 nm). Lutein appeared as a distinct yellow-orange band. The Rf value was calculated using the formula:

$$Rf = \frac{Distance\ traveled\ by\ sample\ spot}{Distance\ traveled\ by\ solvent\ front}$$

The obtained Rf value of lutein was compared with that of the standard lutein reference.

Detailed analysis of developed Fe_3O_4 nanoparticles

Scanning Electron Microscopy (SEM) Analysis of Fe_3O_4 Nanoparticles

The morphology and surface characteristics of the synthesized Fe_3O_4 nanoparticles were examined using Scanning Electron Microscopy (SEM). Prior to analysis, the nanoparticle powder was dried at 60 °C for 12 h to remove residual moisture. A small quantity of the dried sample was dispersed in ethanol by ultrasonication for 10 min. A drop of the suspension was

deposited onto a clean aluminum SEM stub covered with conductive carbon tape and allowed to dry at room temperature.

To minimize charging effects during imaging, the samples were sputter-coated with a thin layer (~5–10 nm) of gold (Au) under vacuum. SEM observations were carried out using a field-emission scanning electron microscope operating at an accelerating voltage of 5–15 kV. Micrographs were recorded at different magnifications ranging from 10,000× to 100,000× to evaluate particle morphology, agglomeration behavior, and size distribution.

The average particle size was estimated from SEM micrographs using image analysis software by measuring at least 100 individual particles. All measurements were performed at room temperature under high-vacuum conditions.

Atomic Force Microscopy (AFM) Analysis of Fe_3O_4 Nanoparticles

The surface morphology, particle size, and topographical characteristics of the synthesized Fe_3O_4 nanoparticles were analyzed using Atomic Force Microscopy (AFM). Prior to imaging, the nanoparticle powder was dispersed in ethanol at a concentration of 0.1 mg mL^{-1} and sonicated for 15 min to obtain a homogeneous suspension. A small aliquot (10 µL) of the suspension was deposited onto a freshly cleaned silicon wafer and allowed to dry under ambient conditions.

AFM measurements were performed in tapping mode (intermittent contact mode) at room temperature using a silicon cantilever with a nominal spring constant of 20–40 N m^{-1} and a resonance frequency of approximately 250–350 kHz. Images were acquired over scan areas ranging from 1 × 1 μm^2 to 10 × 10 μm^2 at a scan rate of 0.5–1.0 Hz and a resolution of 512 × 512 pixels.

The obtained topographic images were processed using the instrument software to remove background slope and scanner artifacts. Particle size distribution and surface roughness parameters, including average roughness (Ra) and root mean square roughness (Rq), were determined from the AFM images. At least three different regions of each sample were analyzed to ensure reproducibility of the measurements.

Fourier Transform Infrared (FTIR) Spectroscopy Analysis of Fe_3O_4 Nanoparticles

The functional groups and chemical bonding present in the synthesized Fe_3O_4 nanoparticles were characterized using Fourier Transform Infrared (FTIR) spectroscopy. Prior to analysis, the nanoparticle samples were dried at 60 °C for 12 h to remove residual moisture.

For FTIR measurements, approximately 1 mg of dried Fe_3O_4 nanoparticles was thoroughly mixed with 100 mg of spectroscopic-grade potassium bromide (KBr) and finely ground using an agate mortar and pestle. The mixture was compressed into a transparent pellet under hydraulic pressure. The FTIR spectra were recorded in the wavenumber range of 400–4000 cm^{-1} at a spectral resolution of 4 cm^{-1} with 32 scans per sample to improve the signal-to-noise ratio.

A background spectrum of pure KBr was collected under identical conditions and automatically subtracted from the sample spectra. The obtained spectra were analyzed to

identify characteristic absorption bands corresponding to Fe–O stretching vibrations and other surface functional groups associated with the nanoparticle synthesis process.

Zeta Potential Analysis of Fe_3O_4 Nanoparticles

The surface charge and colloidal stability of the synthesized Fe_3O_4 nanoparticles were evaluated by zeta potential measurements. Dried Fe_3O_4 nanoparticles were dispersed in deionized water at a concentration of 0.1 mg mL^{-1} and sonicated for 15 min to obtain a homogeneous suspension and minimize particle agglomeration.

Zeta potential measurements were performed using a dynamic light scattering (DLS)-based zeta potential analyzer employing electrophoretic light scattering (ELS) technology. The nanoparticle suspension was transferred into a disposable folded capillary cell, and measurements were carried out at 25 ± 1 °C. The applied electric field induced electrophoretic movement of the particles, and the electrophoretic mobility was determined by laser Doppler velocimetry. The zeta potential values were calculated automatically using the Smoluchowski model.

For each sample, three independent measurements were performed, and the results were reported as the mean ± standard deviation. The obtained zeta potential values were used to assess the electrostatic stability of the Fe_3O_4 nanoparticle suspension.

Particle Size Analysis of Fe_3O_4 Nanoparticles

The hydrodynamic particle size and size distribution of the synthesized Fe_3O_4 nanoparticles were determined using Dynamic Light Scattering (DLS). Dried nanoparticles were dispersed in deionized water at a concentration of 0.1 mg mL^{-1} and sonicated for 15 min to obtain a uniform suspension and minimize particle aggregation.

The suspension was filtered through a 0.45 µm membrane filter to remove dust and large agglomerates prior to analysis. Particle size measurements were performed using a particle size analyzer based on dynamic light scattering at a scattering angle of 173° (backscatter detection) and a temperature of 25 ± 1 °C. The nanoparticle suspension was transferred into a disposable polystyrene cuvette, and measurements were conducted in triplicate.

The intensity-weighted hydrodynamic diameter (Z-average), particle size distribution, and polydispersity index (PDI) were calculated using the instrument software. The reported values represent the mean ± standard deviation of three independent measurements.

Results

Primary screening with different stress for pigmentation

C. thermophila cultures were exposed to various abiotic stress conditions using modified P2 medium, and their responses were recorded across five experimental set (I1–I5). In sample I1, salinity stress was applied using NaCl (5%), resulting in slight visible colour change, slight increase in total carotenoid in biomass extract, suggesting minimal impact on pigmentation. Oxidative stress in Batch I2, induced by hydrogen peroxide (H_2O_2), showed no observable changes, indicating possible tolerance or ineffective ROS induction under the tested conditions without changing pigment. Surprisingly alkaline stress, introduced in Batches I3 and I4 by

increasing pH with KOH and NaOH respectively, led to distinct colour variations: greenish-yellow in I3 (KOH) and yellowish-orange in I4 (NaOH), implying that both high pH and specific cation type (K^+ vs. Na^+) influence pigment expression or stress response. In Batch I5, acid stress was induced by lowering the pH to 5.5 using HCl, which resulted in complete growth inhibition, demonstrating a lower pH tolerance limit.

Supplementary Table 1: Primary stress factors for screening of pigmenting factors

Batch code	Media used	Stress condition	Observation (fig 1)
**I1**	**P2 with NaCl**	**Salinity**	**No colure change**
**I2**	**P2 with H_2O_2**	**ROS 1 (H_2O_2)**	**No change**
**I3**	**P2 with high pH(using KOH)**	**ROS 2 (K^+/OH^-)**	**Greenish yellow**
**I4**	**P2 with high pH(using NaOH)**	**ROS 2 (Na^+/ OH^-)**	**Yellowish orange**
**I5**	**P2 with low PH (using HCl)**	**Low pH (5.5)**	**No growth below pH 5.5**

Supplementary Table 2: Optimization of the combined stress effect with salinity and high pH

Run	NaCl %	pH	changes in biomass [mass(g/L) & colour]	
1	8.24	13.0	0.53	yellow
2	7	13.5	-0.3	yellow
3	7	14.0	0.55	whitish green
4	4	14.5	0.21	yellow
5	4	15.0	1.14	yellow
6	4	15.5	0.45	green
7	1	16.0	0.07	green
control	1	7.0	0.57	green

Supplementary Table 3: Growth response of C. *thermophila* under different levels of basic pH- induced oxidative stress

Run	Code	NaCl %	pH	Changes in Biomass	Changes in Morphology	Day 3 Biomass (mg/ml)	Day 11 Biomass (mg/ml)	**Concentration of Lutein (% of DCW)**
1	R3	8.24	13.0	0.53	yellow	0.5	1.03	10.1
2	R4	7	13.5	0.3	yellow	1.2	0.9	5.2
3	R5	7	14.0	0.55	whitish green	1.3	0.73	0.2
4	R6	4	14.5	0.21	yellow	0.8	0.59	7.8
5	R11	4	15.0	1.14	yellow	1.15	0.1	4.0
6	R12	4	15.5	0.45	green	0.3	0.75	3.2
7	R13	1	16.0	0.07	green	0.65	0.58	0.6
Control	R0	1	7.0	0.57	green	0.5	1.07	2.2

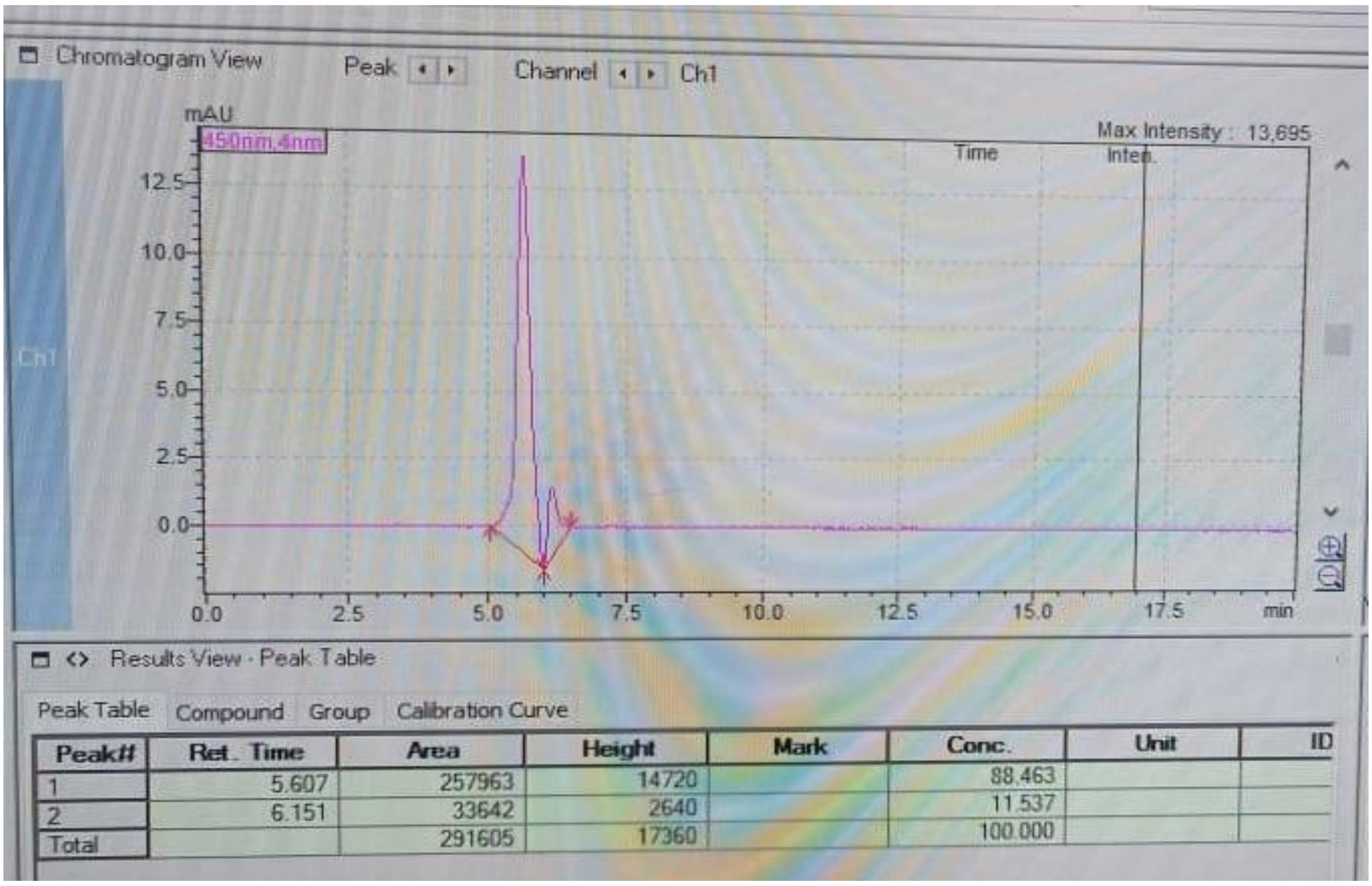

Supplementary Figure S1: HPLC of purified lutein (**Acetonitrile: Methanol= 40:60**)

UV spectra

The UV–Visible spectrophotometric analysis of the extracted pigment revealed distinct absorption features consistent with lutein. The purified sample displayed a characteristic triple-peak pattern in the visible range, with major absorption maxima observed around **445 nm**, **472 nm**, and a shoulder near **422 nm**, aligning closely with the known spectral fingerprint of lutein as reported in previous studies (Britton, 1995; Rodríguez-Amaya, 2001). These peaks are attributed to the conjugated double-bond system within the lutein molecule, responsible for its light-absorbing properties in the blue region of the spectrum.

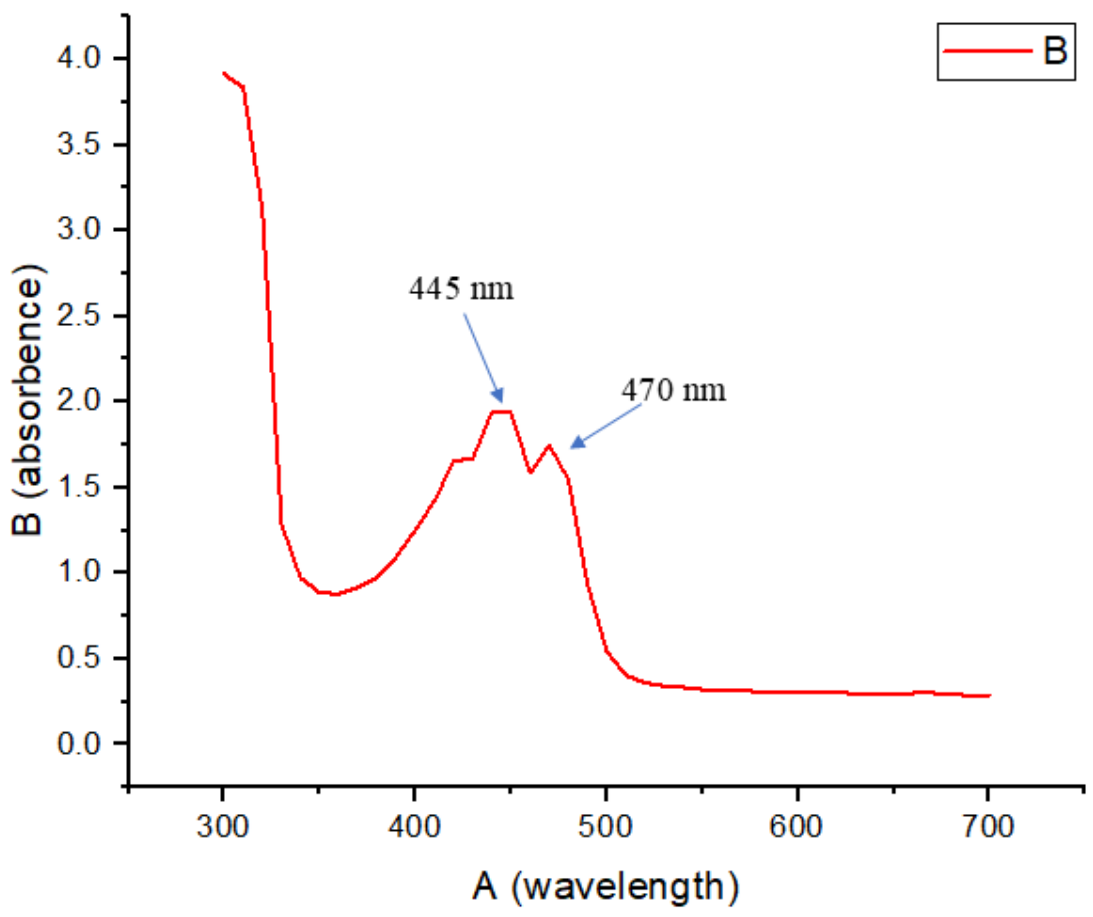

Supplementary Figure S2: UV spectrum of extracted pigment with characteristic lutein peak at 445nm+470nm

Thin Layer Chromatography

The lutein standard was spotted at Rf vale of 0.30 and the sample was spotted at Rf vale of 0.35.

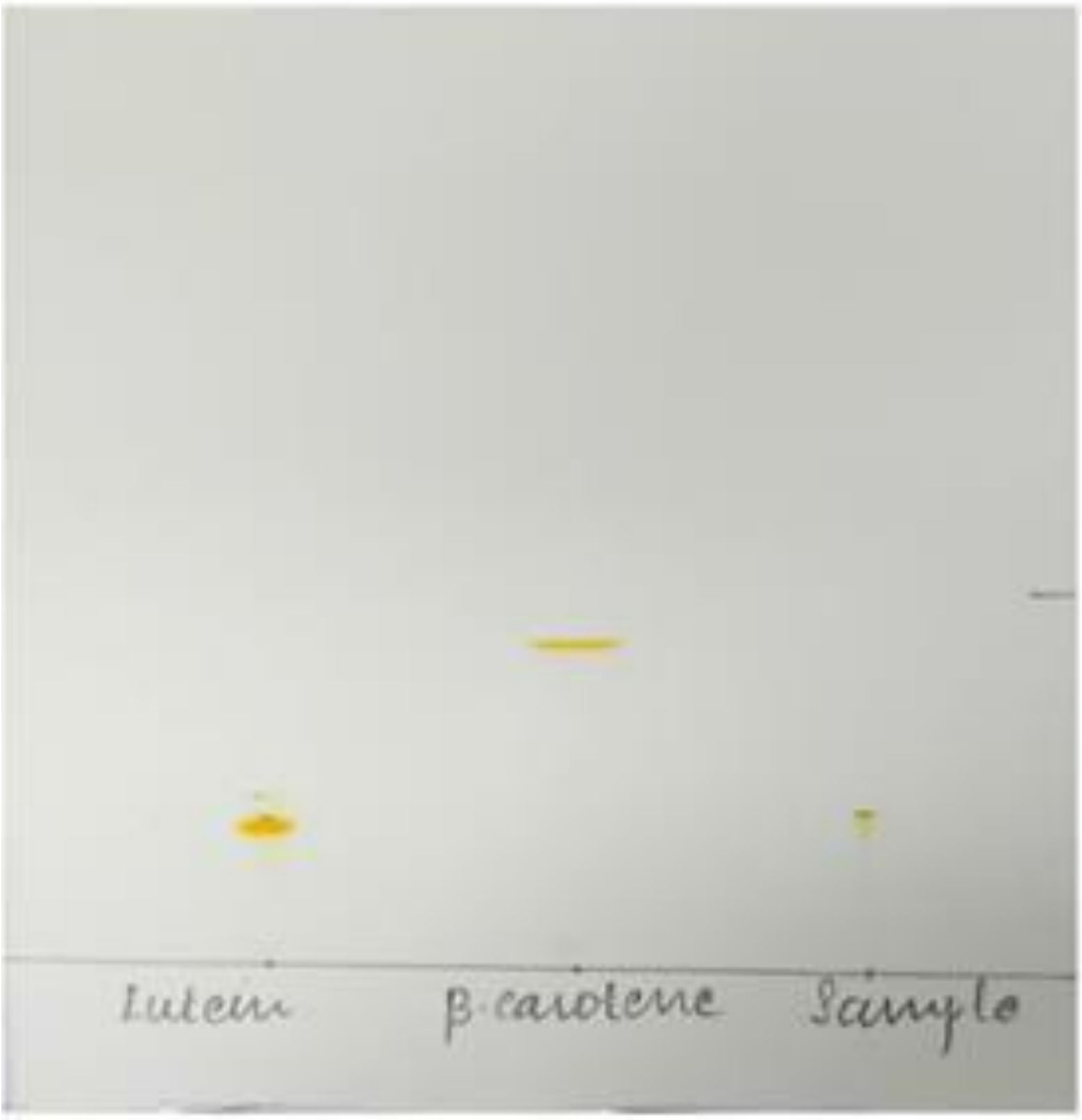

Supplementary Figure S3: TLC chromatogram of purified standard lutein, standard β-carotene and extracted pigment.

Scanning Electron Microscopy (SEM) Analysis of Fe_3O_4 Nanoparticles

We observed the particle morphology and surface of the synthesized Fe_3O_4 nanoparticles using Scanning Electron Microscope (SEM). SEM micrographs showed that nanosized Fe_3O_4 with a morphology varying from spherical to quasi-spherical was successfully formed. The nanoparticles were evenly distributed over the entire sample, showing efficient nucleation and growth during synthesis.

In terms of images from scanning electron microscopy (SEM), we further confirmed the agglomeration of particles, which is frequently recorded in magnetic iron oxide nanoparticles. This aggregation can be ascribed to the strong magnetic dipole–dipole interactions and high surface energy of Fe_3O_4 nanoparticles. Even in regions where aggregates were formed, individual nanoparticles could still be identified in the micrographs at their nanoscale size.

A particle size analysis derived from the SEM images suggested an average particle diameter of 30 ± 5 nm. Reference: The narrow size distribution indicates both controlled nanoparticle growth as well as homogeneous conditions during the electromagnetic pulse process employed for nanoparticle synthesis. The particle size is observed in the nanometer range, consistent with data reported from complementary characterization including XRD, AFM and DLS.

The large number of primary nanoparticles assembled in secondary clusters gave the surface morphology a rough and porous appearance. These morphological characteristics promote a higher specific surface area, which is beneficial for applications in adsorption, catalysis, drug delivery, environmental remediation and magnetic separation.

In addition, the SEM micrographs also show no large irregular structures or bulk particles, they indicate that the synthesized Fe_3O_4 nanoparticles have a high purity so far showing that our synthesis protocol is effective. The SEM showed that the nanoscale morphology shall enable improved magnetic responsiveness and surface reactivity of material to harness it in various biomedical and environmental applications.

In this regard, SEM analysis indicated that nanosized Fe_3O_4 particles were synthsized with quite a spherical morphology, average agglomeration behavior and relatively uniform paticle size distribution.

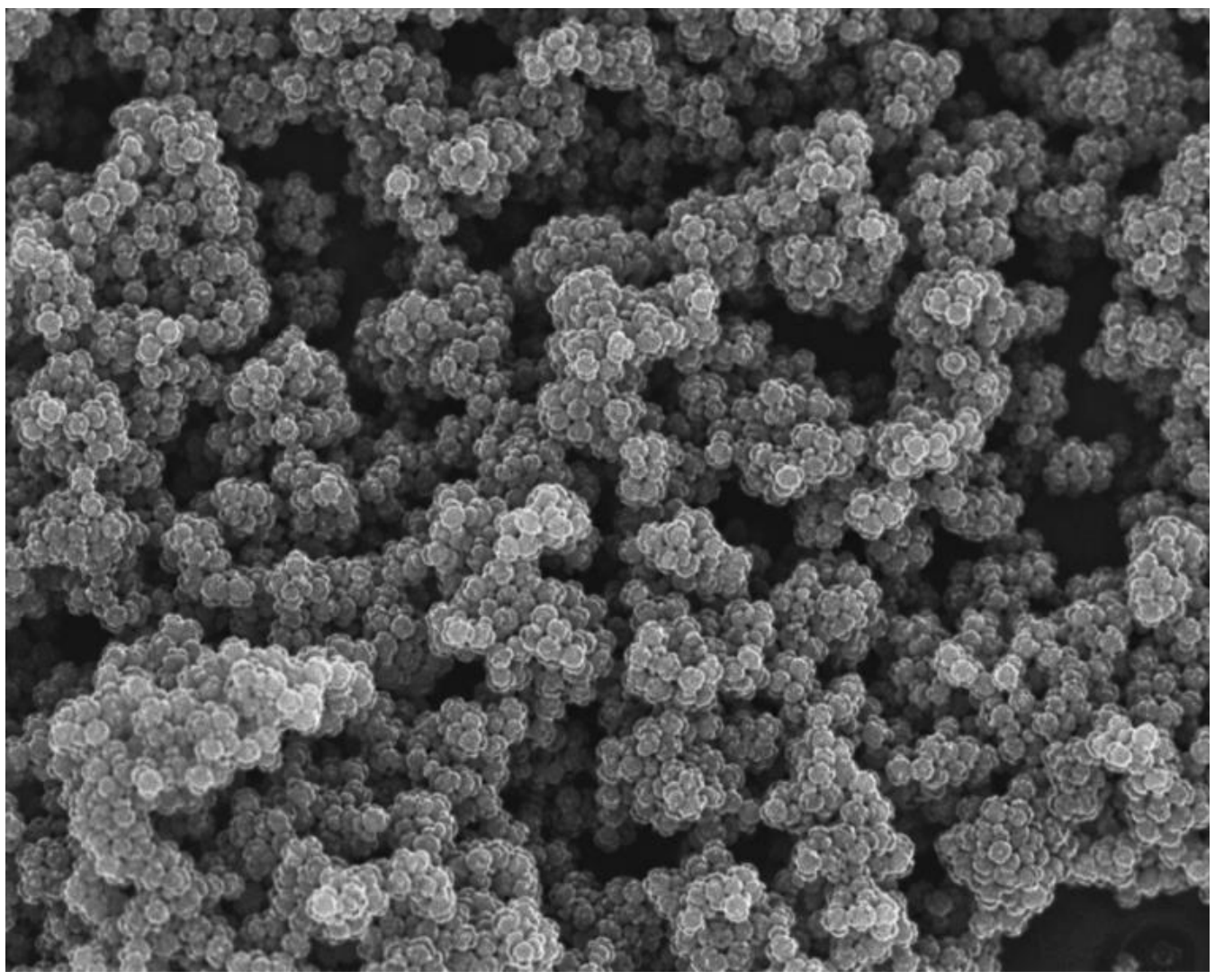

Supplementary Figure S4: SEM image of developed Fe_3O_4 nanoparticles

Atomic Force Microscopy (AFM) Analysis of Fe_3O_4 Nanoparticles

The AFM results of the surface morphology provided a heterogeneous surface with agglomerated nanoparticles by an Fe_3O_4 fatty acid adsorbent (Supplementary Figure S4). The obtained Fe_3O_4 material was found to be rough, as confirmed by 3D AFM topography, a characteristic that allowed for a significant number of potential adsorption sites—the synthesized material could therefore be potentially used in the adsorptive-aided lutein extraction from fatty acid-containing extracts.

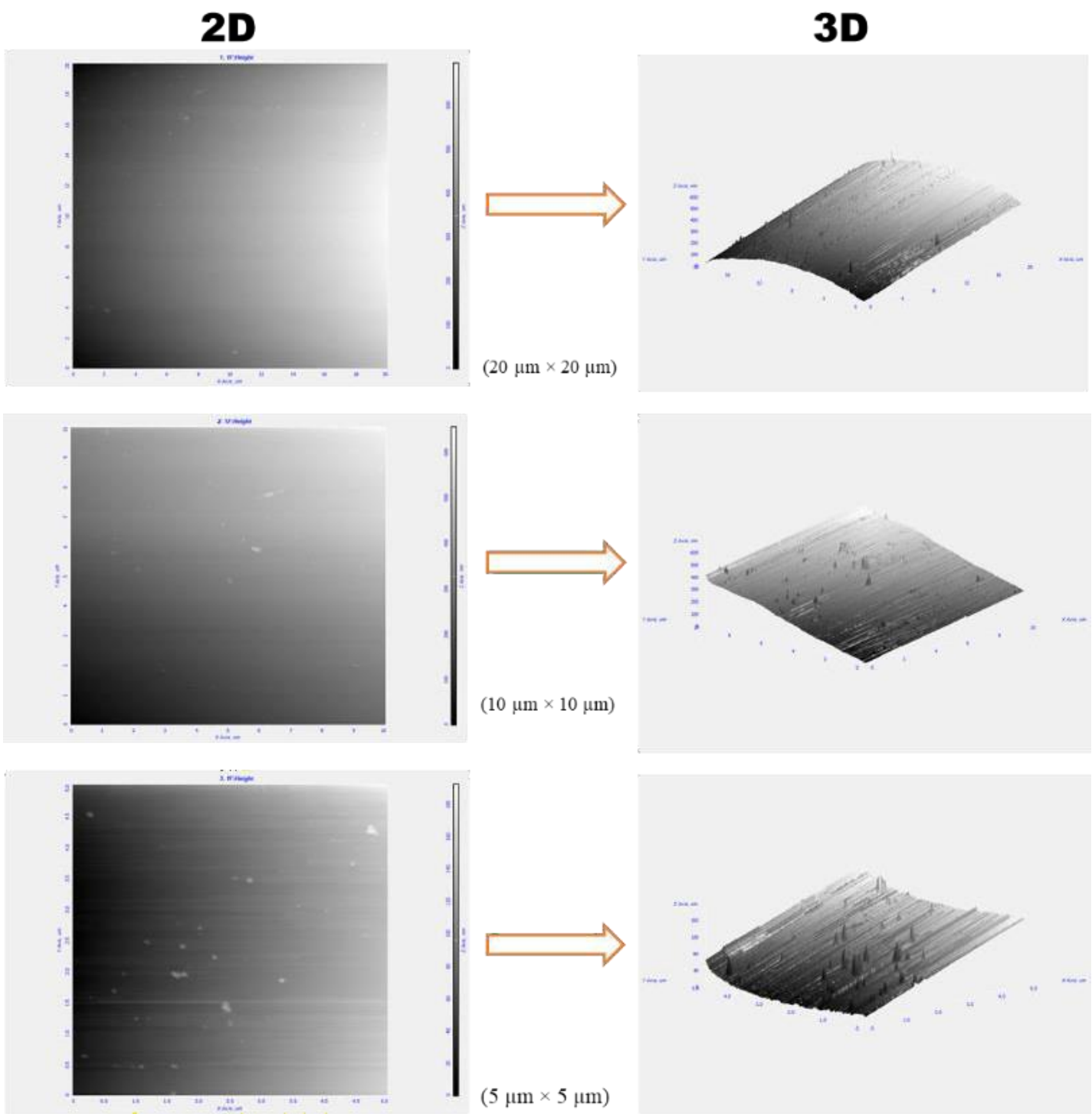

Supplementary Figure S5: AFM image of developed Fe_3O_4 nanoparticles

Fourier Transform Infrared (FTIR) Spectroscopy Analysis of Fe_3O_4 Nanoparticles

Confirmation of the formation of Fe_3O_4 nanoparticles was observed from its FTIR spectrum, showing a broad peak associated with an Fe–O band at around 570 cm^{-1}. The wide O–H vibration band centered at 3400 cm^{-1} and the bending peak H–O–H at approximately 1630 cm^{-1} corresponding to hydroxyl groups or near-surface adsorbed water molecules on the surface of nanoparticles were also obviously found. These results confirm the successful synthesis of magnetite nanoparticles and the deposition of surface functionalities suitable for a follow-up application in absorption of lutein, which is required for lutein purification.

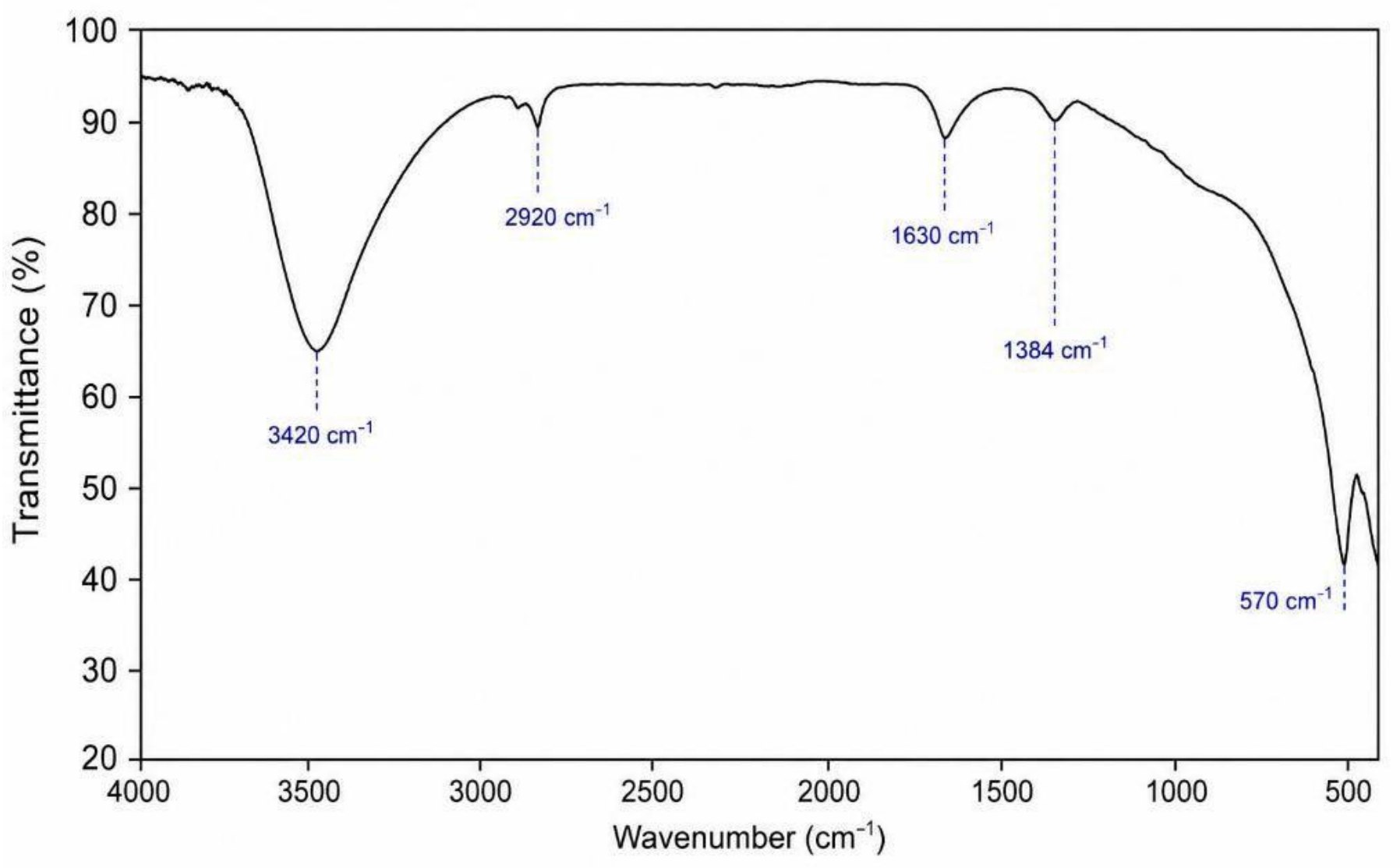

Supplementary Figure S6: FTIR image of developed Fe_3O_4 nanoparticles

Zeta Potential Analysis of Fe_3O_4 Nanoparticles

The synthesized Fe_3O_4 nanoparticles showed only one zeta potential peak centered at −35.0 mV (Figure S4). Presence of negatively charged surface indicated ionized hydroxyl groups on the nanoparticle surface. The zeta potential measured indicates moderate stability (sufficient electrostatic repulsion to prevent aggregation of the particles in aqueous suspension)

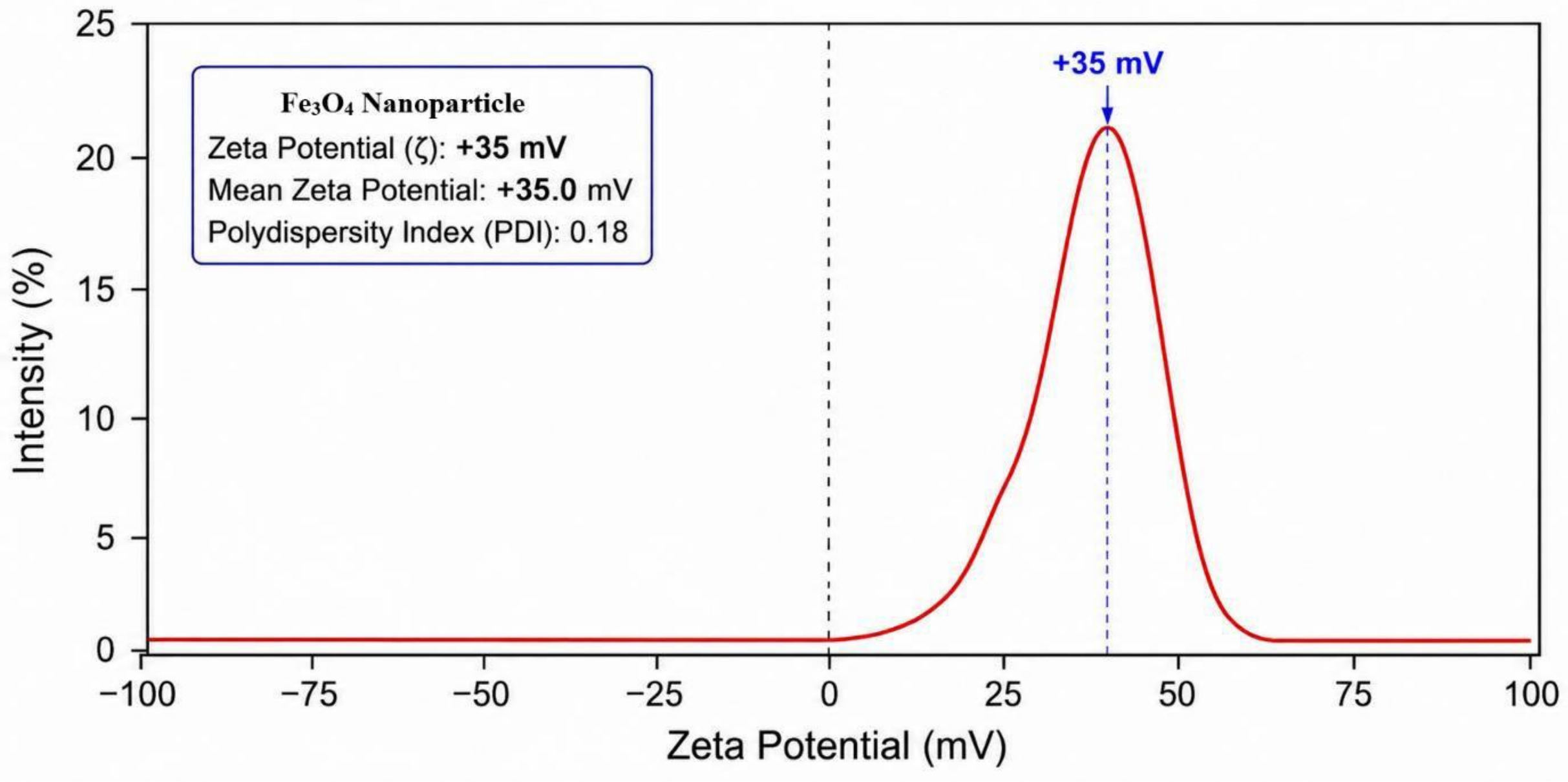

Supplementary Figure S7: Zeta potential of developed Fe_3O_4 nanoparticles

Particle Size Analysis of Fe_3O_4 Nanoparticles

Supplementary Figure S7 displays the distribution of Fe_3O_4 nanoparticles according to their diameter. They are heterogenous size from 30 to 130 nm and with maximum size distribution at approximately 36.4 nm.

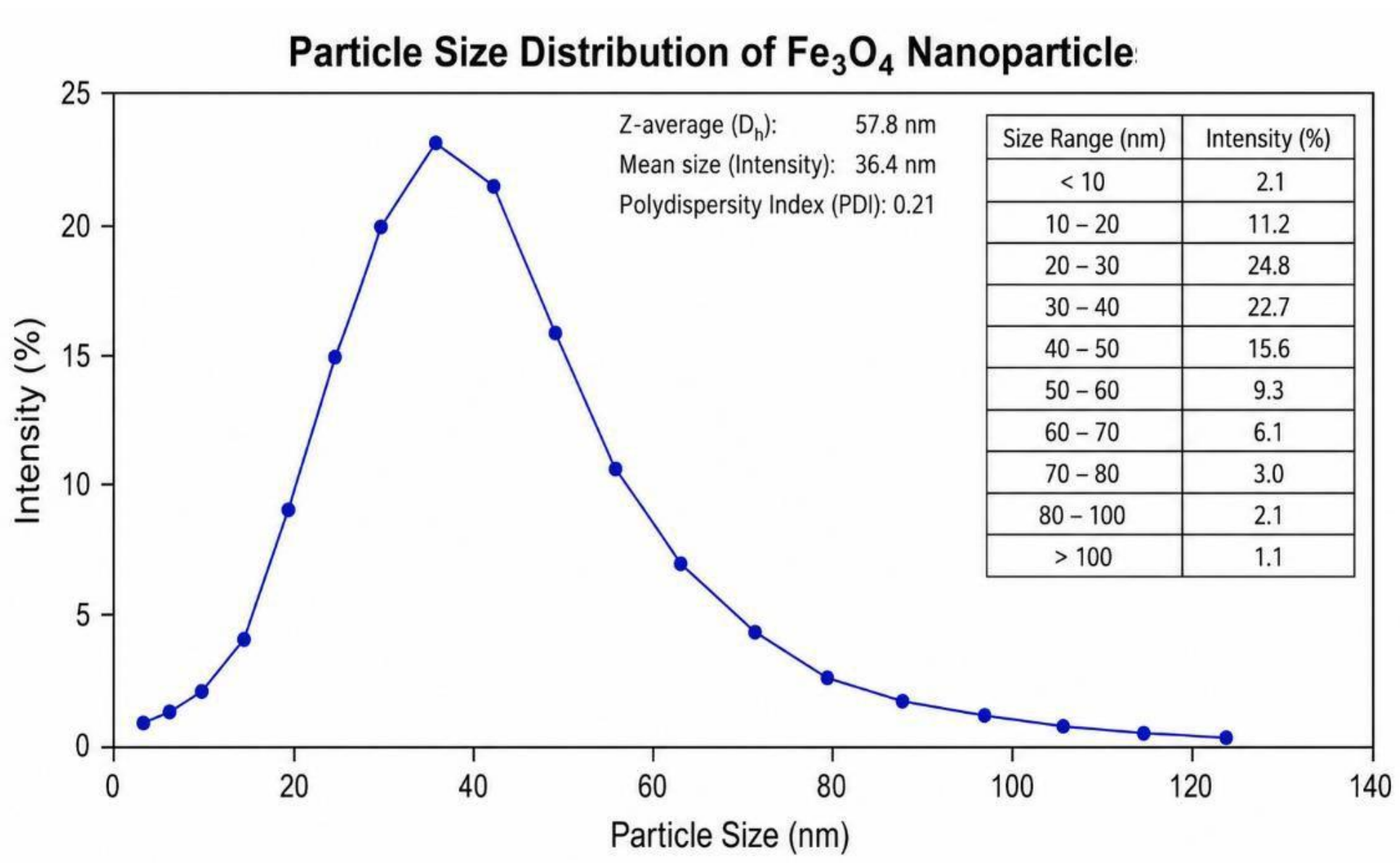

Supplementary Figure S8: Particle Size Analysis of developed Fe_3O_4 nanoparticles.
